# KDM2B is involved in the epigenetic regulation of TGF-β-induced epithelial–mesenchymal transition in lung and pancreatic cancer cell lines

**DOI:** 10.1074/jbc.RA120.015502

**Published:** 2020-12-24

**Authors:** Sasithorn Wanna-Udom, Minoru Terashima, Kusuma Suphakhong, Akihiko Ishimura, Takahisa Takino, Takeshi Suzuki

**Affiliations:** 1 Division of Functional Genomics, Cancer Research Institute, Kanazawa University, Kanazawa Ishikawa, Japan; 2 Division of Education for Global Standard, Institute of Liberal Arts and Science, Kanazawa University, Kanazawa, Ishikawa, Japan

**Keywords:** cancer biology, epithelial–mesenchymal transition, transcription regulation, polycomb repressive complex, histone modification, ChIP, chromatin immunoprecipitation, DMEM, Dulbecco’s modified Eagle’s medium, EMT, epithelial–mesenchymal transition, FBS, fetal bovine serum, GAPDH, glyceraldehyde-3-phosphate dehydrogenase, H2AK119Ub, histone H2A mono-ubiquitinated Lys119, H3K27me3, histone H3 trimethylated Lys27, lncRNA, long noncoding RNA, PcG, Polycomb group, PRC1, polycomb repressive complex-1, QRT-PCR, quantitative reverse transcription polymerase chain reaction, sgRNA, single guide RNA, shRNA, small hairpin RNA, TGF-β, transforming growth factor-beta

## Abstract

Polycomb repressive complex-1 (PRC1) induces transcriptional repression by regulating monoubiquitination of lysine 119 of histone H2A (H2AK119) and as such is involved in a number of biological and pathological processes including cancer development. Previously we demonstrated that PRC2, which catalyzes the methylation of histone H3K27, has an essential function in TGF-β-induced epithelial–mesenchymal transition (EMT) of lung and pancreatic cancer cell lines. Since the cooperative activities of PRC1 and PRC2 are thought to be important for transcriptional repression in EMT program, we investigated the role of KDM2B, a member of PRC1 complex, on TGF-β-induced EMT in this study. Knockdown of *KDM2B* inhibited TGF-β-induced morphological conversion of the cells and enhanced cell migration and invasion potentials as well as the expression changes of EMT-related marker genes. Overexpression of *KDM2B* influenced the expression of several epithelial marker genes such as *CDH1*, *miR200a*, and *CGN* and enhanced the effects of TGF-β. Mechanistic investigations revealed that KDM2B specifically recognized the regulatory regions of *CDH1*, *miR200a*, and *CGN* genes and induced histone H2AK119 monoubiquitination as a component of PRC1 complex, thereby mediating the subsequent EZH2 recruitment and histone H3K27 methylation process required for gene repression. Studies using KDM2B mutants confirmed that its DNA recognition property but not its histone H3 demethylase activity was indispensable for its function during EMT. This study demonstrated the significance of the regulation of histone H2A ubiquitination in EMT process and provided the possibility to develop novel therapeutic strategies for the treatment of cancer metastasis.

Metastasis is the leading cause for mortality of cancer patients. A developmental process termed epithelial–mesenchymal transition (EMT) has been shown to play an essential role in cancer metastasis (1). During EMT, cells lose their epithelial characters such as cell polarity and cell contacts and gain migratory and invasive properties of mesenchymal cells. Physiological activation of EMT can be triggered by extrinsic signals including transforming growth factor-beta (TGF-β) secreted from tumor microenvironment (2). EMT program is orchestrated by the dynamic and reversible changes in epithelial and mesenchymal gene expression (3). One of the most-characterized hallmarks of EMT is the functional loss of E-cadherin, a defining marker of epithelial cells. A cohort of transcription factors such as ZEB and SNAIL family function as molecular switches to activate EMT-inducing transcriptional program through the downregulation of E-cadherin. Furthermore, owing to the plastic nature of EMT, epigenetic regulations including histone modification and DNA methylation are considered as one of the critical mechanisms for EMT (4, 5). Thus, for example, several groups including us have investigated the functional connection between transcriptional repression of *CDH1/E-cadherin* and posttranslational modifications of histones (6, 7, 8).

Posttranslational covalent histone modifications such as methylation, acetylation, ubiquitination, and phosphorylation are central to epigenetic control of gene expression by modulating chromatin structure (9). Lysine (K) methylation of histone H3 (K4, K9, K27, and K36), in particular, has attracted much attention because of its dynamic outcomes of gene expression (10, 11). Methylation of H3K4 and H3K36 is a hallmark of transcriptional active genes, whereas methylation of H3K27 and H3K9 is generally linked to gene repression. These methylations are controlled by the distinct enzymes, namely histone lysine methyltransferases (KMTs) and lysine demethylases (KDMs) called “writers” and “erasers,” respectively. Strict regulation of these enzymes is essential for the biological output, and the deregulated expression of them has been associated with the developmental defects and the pathogenesis of cancer (10, 11, 12). We have previously identified many histone KMTs and KDMs as candidate cancer genes by retroviral insertional mutagenesis in mice and reported that some of them are implicated not only in cancer initiation but also in malignant progression such as cell invasion and EMT (12, 13, 14). Especially, the regulation of histone H3K27 methylation by polycomb repressive complex-2 (PRC2) was shown to be indispensable for EMT-inducing gene expression program in lung and colon cancer cells (15, 16, 17). We have also discovered that target gene-specific recruitment of PRC2 in EMT was regulated by the specific long noncoding RNA (lncRNA) through its “initiator” function (18, 19).

Polycomb group (PcG) proteins are major epigenetic regulators in cell fate, development, and cancer and assembled into two complexes, PRC1 and PRC2 (20, 21). The PRC2 complex, which comprises of EZH2, EED, SUZ12, and other associated proteins, conducts histone H3K27 methylation, thereby adding a repressive mark on the chromatin (22, 23). On the other hand, PRC1 complex catalyzes monoubiquitination of histone H2A on lysine 119 (H2AK119Ub). It is subdivided into two subfamilies of canonical PRC1 and variant PRC1, which include a heterogenous group of several complexes (24). The catalytic core of PRC1 consists of an E3 ubiquitin ligase, RING1A or RING1B, and one of six PCGF proteins. The cooperative activities of PRC1 and PRC2 are thought to be important for PcG-mediated transcriptional repression (25). However, the molecular mechanism by which PRC1 and PRC2 recognize their target genes and induce repressive chromatin state seems to be different between canonical and variant PRC1. The canonical PRC1 containing CBX proteins can recognize H3K27me3 and occupy the chromatin modified by PRC2 to mediate gene repression. In contrast, variant PRC1 complexes have been proposed to contribute to transcriptional repression of the target genes in the absence of pre-existing H3K27me3 (26, 27).

In this study, we tried to clarify the involvement of PRC1 components in the regulation of TGF-β-induced EMT of A549 lung cancer and Panc1 pancreatic cancer cell lines. We found that KDM2B, one of the members of variant PRC1 complex, was indispensable for EMT as an essential regulator of transcriptional repression of several epithelial marker genes including *CDH1*, *miR200a*, and *CGN*. Mechanistically, KDM2B was shown to associate with the regulatory regions of these epithelial genes and epigenetically repress the expression through histone H2A ubiquitination, indicating the importance of KDM2B-mediated PRC1 activity during EMT process.

## Results

### KDM2B is indispensable for TGF-*β*-induced EMT process in A549 lung cancer and Panc1 pancreatic cancer cells

In order to find the PRC1 components involved in TGF-β-induced EMT of cancer cells, we tried to perform a candidate gene approach. Namely, we examined the effect of downregulation of each PRC1-related gene by CRISPR/Cas9 system on the changes in EMT character of A549 lung cancer cell line (Fig. S1). We picked up 23 PRC1-related genes encoding the core subunits (RING1A/1B and six PCGF proteins) and the accessary proteins based on the previous report (28). Five different sgRNAs for each candidate gene were introduced into the doxycycline-inducible Cas9-expressing A549 cells (A549/Cas9h, see experimental procedures) by lentiviral infection. The infected cells were treated with doxycycline and the knockdown efficiency of each gene was examined by quantitative RT-PCR (QRT-PCR). The expressions of all 23 PRC1-related genes were significantly decreased after doxycycline treatment (Fig. S2*A*). For several genes that showed relatively small reduction at mRNA levels, we performed immunoblotting to see the knockdown effects at protein levels. We confirmed that RING1A, RING1B, PHC1, SKP1, KDM2B, and RYBP proteins were efficiently downregulated by the sgRNAs (Fig. S2*B*). To find the potential involvement of each PRC1 member in EMT process, we analyzed the expression of *CDH1/E-cadherin*, an epithelial marker gene, in the knockdown cells by QRT-PCR (Fig. S2*C*). The expression of *CDH1* was significantly affected by the sgRNAs for five PRC1-related genes, *PCGF1*, *BCOR*, *SKP1*, *KDM2B*, and *RYBP* (Fig. S2*C*). Among them, we decided to focus on *KDM2B* gene as a candidate in this study because its downregulation caused the most remarkable changes in *CDH1* expression in A549 cells.

To validate the involvement of *KDM2B* in TGF-β-induced EMT process, we used two different cancer cell lines, A549 lung cancer and Panc1 pancreatic cancer cells, and treated them with TGF-β. QRT-PCR revealed that *KDM2B* expression was increased by TGF-β in both cells, suggesting its possible involvement (Fig. S3, *A*–*B*). We also detected the increase of endogenous KDM2B protein by TGF-β with the immunoprecipitation followed by immunoblotting (Fig. S3, *E*–*F*), although we did not detect endogenous KDM2B protein with our KDM2B antibody by simple immunoblotting (See Fig. S4, *C*–*D*). Then we examined the effects of *KDM2B* knockdown in A549 and Panc1 cells to clarify its function in EMT process. We used two different shRNAs (*KDM2B* shRNA#1 and #2) and confirmed the downregulation of *KDM2B* even in the presence of TGF-β in both cells by QRT-PCR and immunoprecipitation (Fig. S3, *A*, *B*, *E* and *F*). We also observed that both *KDM2B* shRNAs caused the same effects in the expression of *CDH1* gene in both cells (Fig. S3, *C*–*D*), and therefore the data of *KDM2B* shRNA#2 were shown as a representative result.

**Figure 1:**
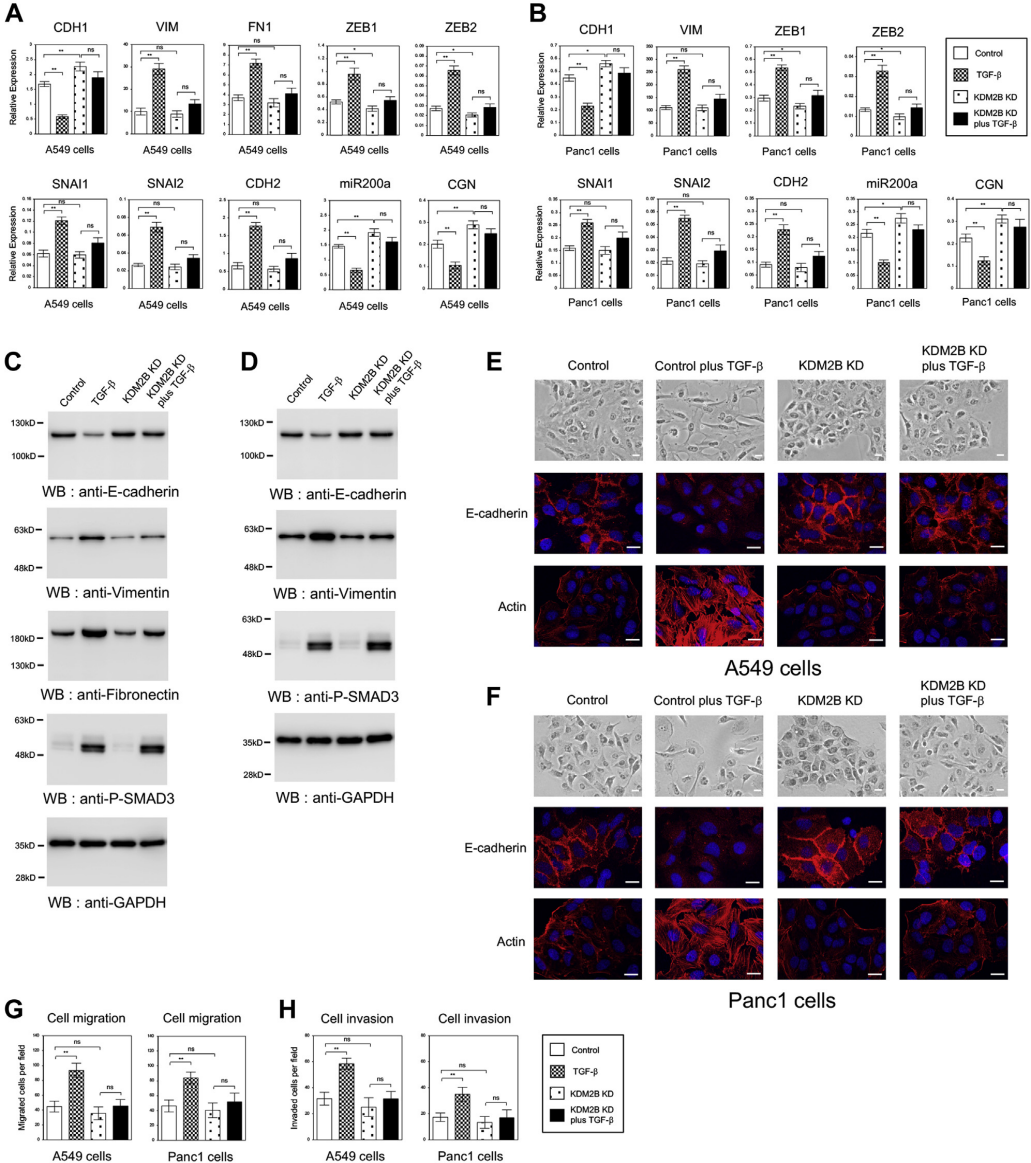
**Knockdown of *KDM2B* antagonized TGF-β-induced EMT phenotypes in A549 lung cancer and Panc1 pancreatic cancer cells.***A*–*B*, knockdown of *KDM2B* affected the changes in expression of EMT-related marker genes induced by TGF-β. QRT-PCR was performed to detect the expression of *CDH1*, *VIM*, *FN1*, *ZEB1*, *ZEB2*, *SNAI1*, *SNAI2*, *CDH2*, *miR200a*, and *CGN* in the control or *KDM2B* knockdown (KD) A549 (*A*) and Panc1 (*B*) cells with or without TGF-β for 24 h (n = 3) (∗∗*p* < 0.01; ∗*p* < 0.05; ns, not significant). *C*–*D*, immunoblotting of E-cadherin, Fibronectin, Vimentin, phosphorylated SMAD3 (P-SMAD3), and GAPDH proteins using the corresponding antibodies in A549 (*C*) and Panc1 (*D*) cells. *E*–*F*, *KDM2B* knockdown inhibited TGF-β-induced morphological changes of A549 (*E*) and Panc1 (*F*) cells. The cells were stained with crystal violet (*upper*), with anti-E-cadherin antibody and DAPI (*middle*), or with TRITC-phalloidin and DAPI (*lower*). Scale bars: 10 μm. *G*–*H*, *KDM2B* knockdown inhibited TGF-β-dependent increase of migrated cells (*G*) or invaded cells (*H*) through the filter. A549 and Panc1 cells that migrated or invaded through the filter were fixed, stained, and counted (n = 8) (∗∗*p* < 0.01; ns, not significant).

During EMT, epithelial cells acquire a mesenchymal phenotype through downregulating epithelial cell markers such as *CDH1*, *miR200a* (*microRNA-200a*), and *CGN/Cingulin* and upregulating mesenchymal cell markers including *VIM*, *FN1*, *ZEB1*, *ZEB2*, *SNAI1*, *SNAI2*, and *CDH2* (3, 29). We examined the expression levels of them by QRT-PCR. *KDM2B* knockdown significantly increased the expression of epithelial marker genes such as *CDH1*, which was consistent with the above results in Fig. S1, and inhibited its transcriptional repression mediated by TGF-β in A549 and Panc1 cells (Fig. 1, *A*–*B*). For the mesenchymal markers other than *ZEB* family, *KDM2B* knockdown by itself had no influence on their expression (Fig. 1, *A*–*B*), although *FN1* expression was too low to be analyzed in Panc1 cells. For *ZEB1* and *ZEB2*, the targets of *miR200a*, *KDM2B* knockdown itself slightly but significantly reduced their expressions, which was correlated well with the upregulation of *miR200a* (Fig. 1, *A*–*B*). Importantly, *KDM2B* knockdown prevented TGF-β-dependent increase of the expression of mesenchymal marker genes in the cells (Fig. 1, *A*–*B*). We could also confirm these inhibitory effects of *KDM2B* knockdown on the protein expression of E-cadherin, Vimentin, and Fibronectin during TGF-β-induced EMT in A549 and Panc1 cells (Fig. 1, *C*–*D*). To examine the possibility that *KDM2B* knockdown would influence TGF-β signaling pathway in the cells, the status of phosphorylated SMAD3 protein was analyzed (2). The phosphorylation levels of SMAD3 in the presence of TGF-β were similar in the control cells and the *KDM2B* knockdown cells (Fig. 1, *C*–*D*), indicating that TGF-β signaling pathway would not be impaired by *KDM2B* knockdown. These results suggested that *KDM2B* might be involved in TGF-β-dependent transcriptional regulation of EMT-related genes in lung and pancreatic cancer cells without influencing the direct activation of transcription factors by TGF-β.

Next we examined the cell morphologies and the state of E-cadherin and actin in A549 and Panc1 cells associated with EMT phenotypes. Control cells showed scattered, elongated, or enlarged cell shapes, lost E-cadherin staining on the cell membrane, and caused well-organized formation of actin stress fibers after TGF-β treatment (Fig. 1, *E*–*F*). *KDM2B* knockdown itself seemed to enhance cell adhesion and increased E-cadherin staining slightly (Fig. 1, *E*–*F*), which was consistent with the results of QRT-PCR and immunoblotting (Fig. 1, *A*–*D*). In addition, *KDM2B* knockdown markedly suppressed the ability of TGF-β to induce mesenchymal morphology, E-cadherin downregulation, and actin remodeling (Fig. 1, *E*–*F*). These results indicated that *KDM2B* knockdown antagonized TGF-β-induced EMT phenotypes of A549 and Panc1 cells such as morphological changes and cytoskeletal rearrangements.

We further measured cell motility and cell invasion by the transfilter assays to observe the effects of *KDM2B* knockdown in EMT-associated phenotypes. In line with the inhibition of EMT, *KDM2B* knockdown significantly attenuated TGF-β-induced cell migration (Fig. 1*G*) and cell invasion (Fig. 1*H*) in A549 and Panc1 cells. Taken together, we concluded that *KDM2B* was indispensable for TGF-β-induced EMT process in A549 lung cancer and Panc1 pancreatic cancer cells.

### Overexpression of KDM2B partially influenced the gene expression program in EMT.

**Figure 2:**
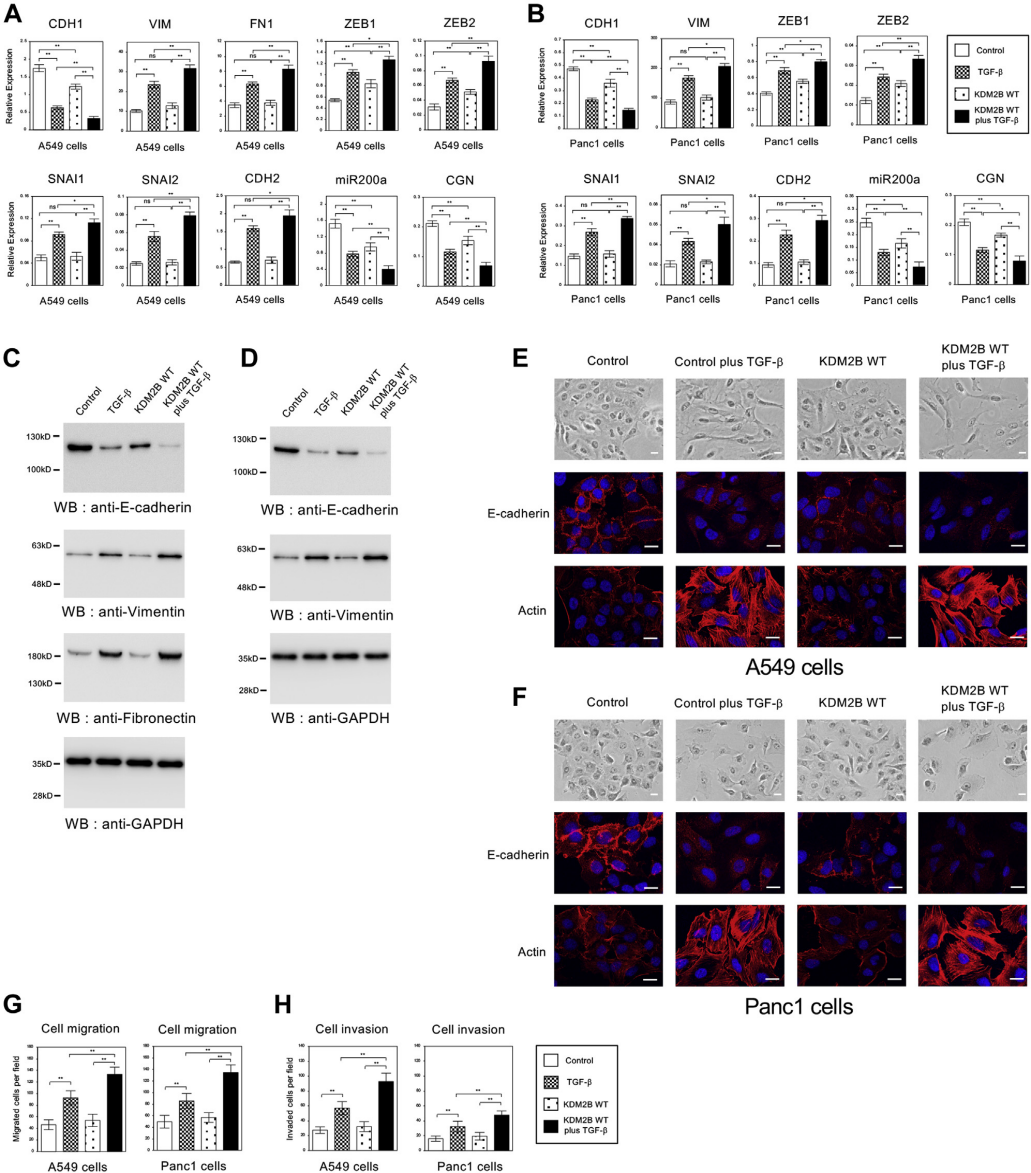
**Overexpression of *KDM2B* repressed the expression of several epithelial marker genes but did not lead to complete EMT phenotypes in A549 and Panc1 cells.***A*–*B*, QRT-PCR analysis of *CDH1*, *VIM*, *FN1*, *ZEB1*, *ZEB2*, *SNAI1*, *SNAI2*, *CDH2*, *miR200a*, and *CGN* in A549 (*A*) and Panc1 (*B*) cells infected with the control retrovirus or the retrovirus expressing wild-type *KDM2B* (WT) with or without TGF-β for 24 h (n = 3) (∗∗*p* < 0.01; ∗*p* < 0.05; ns, not significant). *C*–*D*, immunoblotting of E-cadherin, Fibronectin, Vimentin, and GAPDH proteins in A549 (*C*) and Panc1 (*D*) cells with *KDM2B* overexpression. *E*–*F*, cell morphological changes induced by *KDM2B* overexpression in A549 (*E*) and Panc1 (*F*) cells. The cells were stained similarly in the case of Figure 1. Scale bars: 10 μm. *G*–*H*, cell migration (*G*) or cell invasion (*H*) activities of A549 and Panc1 cells with *KDM2B* overexpression (n = 8) (∗∗*p* < 0.01).

The findings above triggered us to further investigate the effects of *KDM2B* overexpression in EMT process of A549 and Panc1 cells. Both cells were infected with the retroviruses expressing human full-length *KDM2B* (WT), and its overexpression was confirmed by QRT-PCR (Fig. S4, *A*–*B*) and immunoblotting (Fig. S4, *C*–*D*). Then we assessed the expression of EMT marker genes in A549 and Panc1 cells. Overexpression of *KDM2B* led to significant decrease in the expression of epithelial genes such as *CDH1*, *miR200a*, and *CGN* and significant increase in *ZEB* family expression possibly regulated by *miR200a* (Fig. 2, *A*–*B*). However, *KDM2B* overexpression had no effect in the expression of mesenchymal genes other than *ZEB* family (Fig. 2, *A*–*B*). In the presence of TGF-β, *KDM2B* overexpression potentiated the effects of TGF-β in the expression of these EMT marker genes (Fig. 2, *A*–*B*). We also confirmed the similar effects of *KDM2B* overexpression in protein expression of E-cadherin, Vimentin, and Fibronectin in both cells (Fig. 2, *C*–*D*). These results indicated that *KDM2B* overexpression by itself could repress the expression of several epithelial marker genes but not affect the expression of most of mesenchymal markers in A549 and Panc1 cells.

For the cell morphologies of A549 and Panc1 cells, cells overexpressing *KDM2B* revealed slight scattering compared with the control cells, but the effect was not significant (Fig. 2, *E*–*F*). More importantly, *KDM2B* overexpression reduced E-cadherin staining but failed to induce well-organized actin stress fiber formation (Fig. 2, *E*–*F*). We could observe that *KDM2B* overexpression slightly enhanced EMT-related morphological changes of the cells in the presence of TGF-β (Fig. 2, *E*–*F*). The observed cell phenotypes caused by *KDM2B* overexpression were closely correlated with its effects on gene expression (Fig. 2, *A*–*D*). Cell motility and invasion assays in A549 and Panc1 cells also revealed that *KDM2B* overexpression showed no significant change but enhanced the effect of TGF-β (Fig. 2, *G*–*H*). These results suggested that *KDM2B* overexpression by itself could induce the downregulation of several epithelial marker genes, which is one of the critical events in EMT, but might not proceed EMT process completely.

### Overexpression of KDM2B influenced histone H2A ubiquitination and H3 methylation on the regulatory regions of several epithelial genes such as CDH1, miR-200a, and CGN

**Figure 3:**
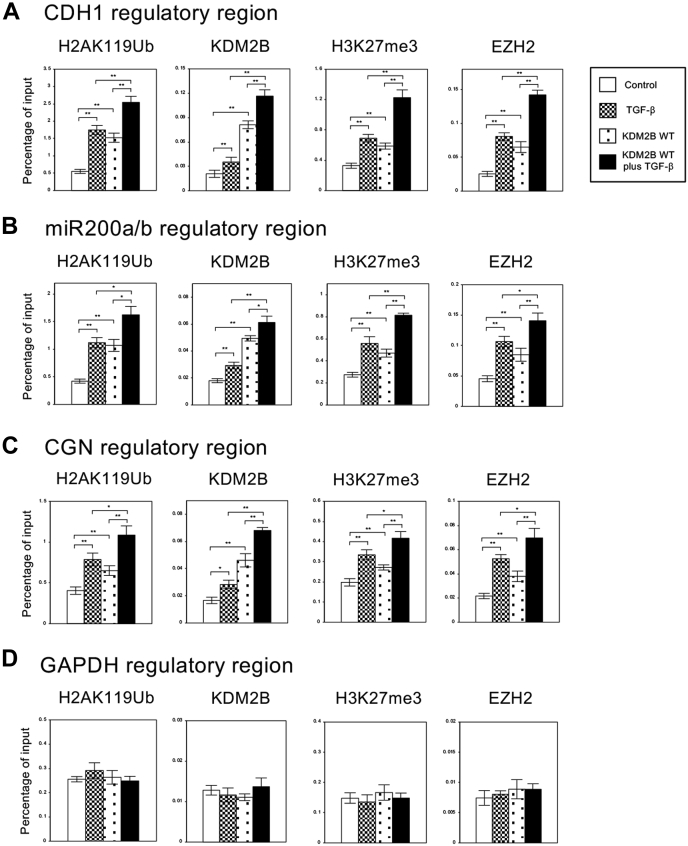
***KDM2B* influenced the regulation of histone H2A ubiquitination and H3 methylation on the regulatory regions of several epithelial marker genes in A549 cells.** A549 cells were infected with the control retrovirus or the retrovirus expressing wild-type *KDM2B* (WT) with or without TGF-β treatment. ChIP analyses of H2AK119Ub, KDM2B, H3K27me3, and EZH2 on the regulatory regions of *CDH1* (*A*), *miR200a/b* (*B*), *CGN* (*C*), and *GAPDH* genes (*D*) in A549 cells are shown. The occupancies of ubiquitinated histones, KDM2B, methylated histones or EZH2 proteins on the regions were analyzed by quantitative PCR. Percentage enrichment over input chromatin DNA was presented (n = 3) (∗∗*p* < 0.01; ∗*p* < 0.05).

For the transcriptional repression of these epithelial genes by KDM2B, we hypothesized that KDM2B function as a component of PRC1 complex might be important. Since PRC1 is involved in monoubiquitination of lysine 119 of histone H2A (H2AK119) for gene repression (20, 21), we first performed chromatin immunoprecipitation (ChIP) assay to analyze the status of histone H2A ubiquitination on the regulatory regions of *CDH1*, *miR200a/b*, and *CGN* genes in A549 cells. TGF-β treatment resulted in a clear increase of ubiquitinated H2AK119 (H2AK119Ub) on these regulatory regions and overexpression of *KDM2B* enhanced H2AK119Ub in the presence or absence of TGF-β (Fig. 3, *A*–*C*). In addition, we could detect the recruitment of endogenous and exogenously expressed KDM2B on these regulatory regions, and it was stimulated by TGF-β treatment (Fig. 3, *A*–*C*). These states of H2AK119 ubiquitination and KDM2B recruitment on these regulatory regions were well correlated with the transcriptional repression of these genes (Fig. 2*A*). Interestingly, we could also observe the changes in histone H3K27 trimethylation (H3K27me3) and EZH2 recruitment induced by *KDM2B* overexpression. As reported previously, TGF-β caused the increase of H3K27me3 and EZH2 binding on the regulatory regions of these epithelial genes, which was closely correlated with transcriptional repression (Fig. 3, *A*–*C*) (15, 16). *KDM2B* overexpression by itself increased H3K27me3 levels and EZH2 binding and further enhanced them in the presence of TGF-β (Fig. 3, *A*–*C*). These results suggested that KDM2B or KDM2B-mediated H2A ubiquitination might enhance the EZH2 recruitment for H3K27 methylation on these regulatory regions. On the regulatory region of unrelated *GAPDH* gene, we did not observe any changes in H2AK119Ub, KDM2B, H3K27me3, and EZH2 signals by *KDM2B* overexpression (Fig. 3*D*). These results indicated that KDM2B could interact with the chromatin regions of the specific target genes including *CDH1*, *miR200a/b*, and *CGN* and mediate H2A ubiquitination, EZH2 recruitment, and H3K27 methylation. Thus we concluded that KDM2B function as a component of PRC1 complex was involved in the transcriptional repression of these epithelial genes.

### Combined knockdown of RING1A and RING1B inhibited the expression changes of the epithelial genes similarly in the case of KDM2B knockdown during EMT

**Figure 4:**
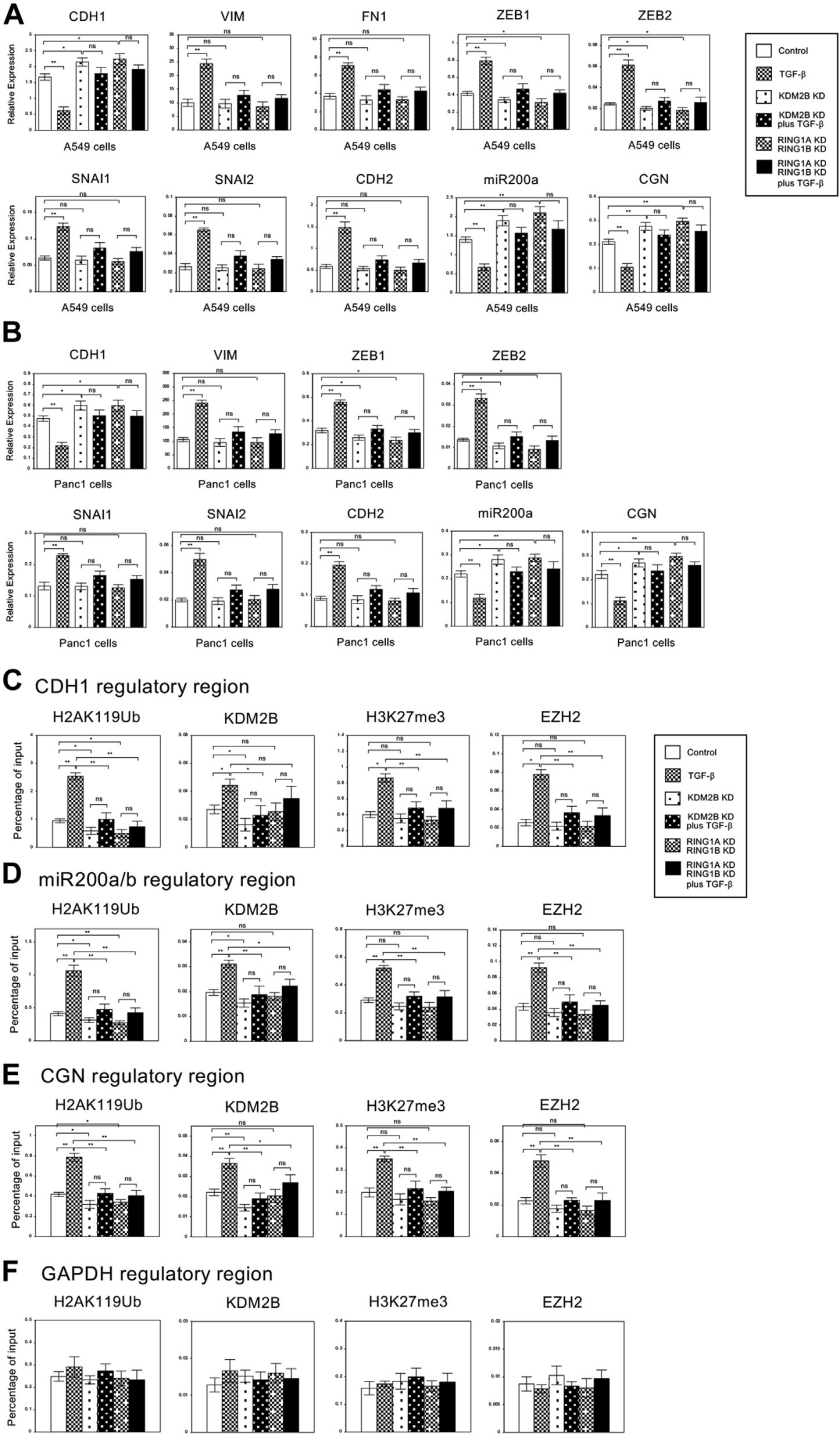
**Combined knockdown of *RING1A* and *RING1B* showed similar inhibitory effects with *KDM2B* knockdown on TGF-β-dependent transcriptional regulation during EMT.***A*–*B*, combined knockdown of *RING1A* and *RING1B* affected the changes in expression of EMT-related marker genes induced by TGF-β. QRT-PCR was performed to detect the expression of *CDH1*, *VIM*, *FN1*, *ZEB1*, *ZEB2*, *SNAI1*, *SNAI2*, *CDH2*, *miR200a*, and *CGN* in the control, *KDM2B*, or *RING1A/RING1B* knockdown (KD) A549 (*A*) and Panc1 (*B*) cells with or without TGF-β for 24 h (n = 3) (∗∗*p* < 0.01; ∗*p* < 0.05; ns, not significant). *C*–*F*, ChIP analyses of H2AK119Ub, KDM2B, H3K27me3, and EZH2 on the regulatory regions of *CDH1* (*C*), *miR200a/b* (*D*), *CGN* (*E*), and *GAPDH* genes (*F*) in A549 cells with *KDM2B* or *RING1A/RING1B* knockdown (n = 3) (∗∗*p* < 0.01; ∗*p* < 0.05; ns, not significant).

Since a variant PRC1 complex contains KDM2B and RING1A or RING1B, an essential catalytic subunit of PRC1, we tried to examine the involvement of RING1A and RING1B during TGF-β-induced EMT (24, 26, 27). The expression of *RING1A* and *RING1B* was shown significantly upregulated in response to TGF-β in A549 and Panc1 cells (Fig. S5, *A*–*D*), suggesting their possible involvement in EMT. Then we performed knockdown experiments for *RING1A* and *RING1B* during EMT process. We showed the data using *RING1A* shRNA#1 and *RING1B* shRNA#2 as a representative result (See Methods and Fig. S5, *A*–*D*). When the expression of either *RING1A* or *RING1B* was knocked down in A549 and Panc1 cells, we did not see any changes in *CDH1* expression during TGF-β-induced EMT (Fig. S6, *A*–*B*). These results were consistent with the results of CRISPR/Cas9 screening showing that *CDH1* expression was not significantly changed by the sgRNAs of either *RING1A* or *RING1B* (Fig. S2*C*). This might be due to the compensatory effect of redundant function of RING1A and RING1B. Thus, we tried to knock down both *RING1A* and *RING1B* by the coinfection of lentiviruses expressing each shRNA, and the reduced expression of each gene was confirmed by QRT-PCR (Fig. S7). With the combined knockdown of *RING1A* and *RING1B* (indicated as *RING1A/1B*), we could detect the clear effects on EMT-related gene expression in A549 and Panc1 cells (Fig. 4, *A*–*B*). Combined knockdown of *RING1A*/*1B* increased the expression of epithelial genes such *CDH1*, *miR200a*, and *CGN* and inhibited its transcriptional repression mediated by TGF-β similarly in the case of *KDM2B* knockdown (Fig. 4, *A*–*B*). *RING1A*/*1B* knockdown itself decreased *ZEB* family expression significantly and had no influence on the expression of other mesenchymal genes. However, *RING1A*/*1B* knockdown counteracted the effect of TGF-β on all these mesenchymal gene expressions in both cells (Fig. 4, *A*–*B*). These results indicated that RING1A and RING1B, catalytic subunits of PRC1, were also indispensable for the downregulation of these several epithelial marker genes during TGF-β-induced EMT. The similar phenotypes of *RING1A*/*1B* and *KDM2B* knockdown suggested the important role of a variant PRC1 complex containing RING1A/1B and KDM2B in EMT.

Next we examined how KDM2B and RING1A/1B affected the expression of epithelial genes such as *CDH1*, *miR200a*, and *CGN* during EMT of A549 cells by the ChIP experiment (Fig. 4, *C*–*E*). *KDM2B* or *RING1A*/*1B* knockdown by itself caused significant decrease of H2AK119Ub on these regulatory regions and antagonized TGF-β-effect on the increase of H2AK119Ub (Fig. 4, *C*–*E*), indicating the essential function of KDM2B and RING1A/1B in histone H2A ubiquitination during EMT. KDM2B recruitment on these regulatory regions was clearly reduced by *KDM2B* knockdown, but not significantly affected by *RING1A*/*1B* knockdown (Fig. 4, *C*–*E*). More importantly, TGF-β-dependent increase of H3K27me3 levels and EZH2 binding on these epithelial genes was inhibited by either *KDM2B* or *RING1A*/*1B* knockdown (Fig. 4, *C*–*E*). These changes were specific to the regulatory regions of *CDH1*, *miR200a*, and *CGN* genes and not observed at the regulatory region of unrelated *GAPDH* gene (Fig. 4*F*). These results suggested that the ubiquitination of H2AK119 on the regulatory regions of these epithelial genes by KDM2B and RING1A/1B was indispensable for the subsequent increase of EZH2 binding and H3K27me3 levels, which resulted in the transcriptional repression of these genes during TGF-β-induced EMT.

### DNA recognition property but not histone demethylase activity was indispensable for KDM2B function in transcriptional regulation during EMT

**Figure 5:**
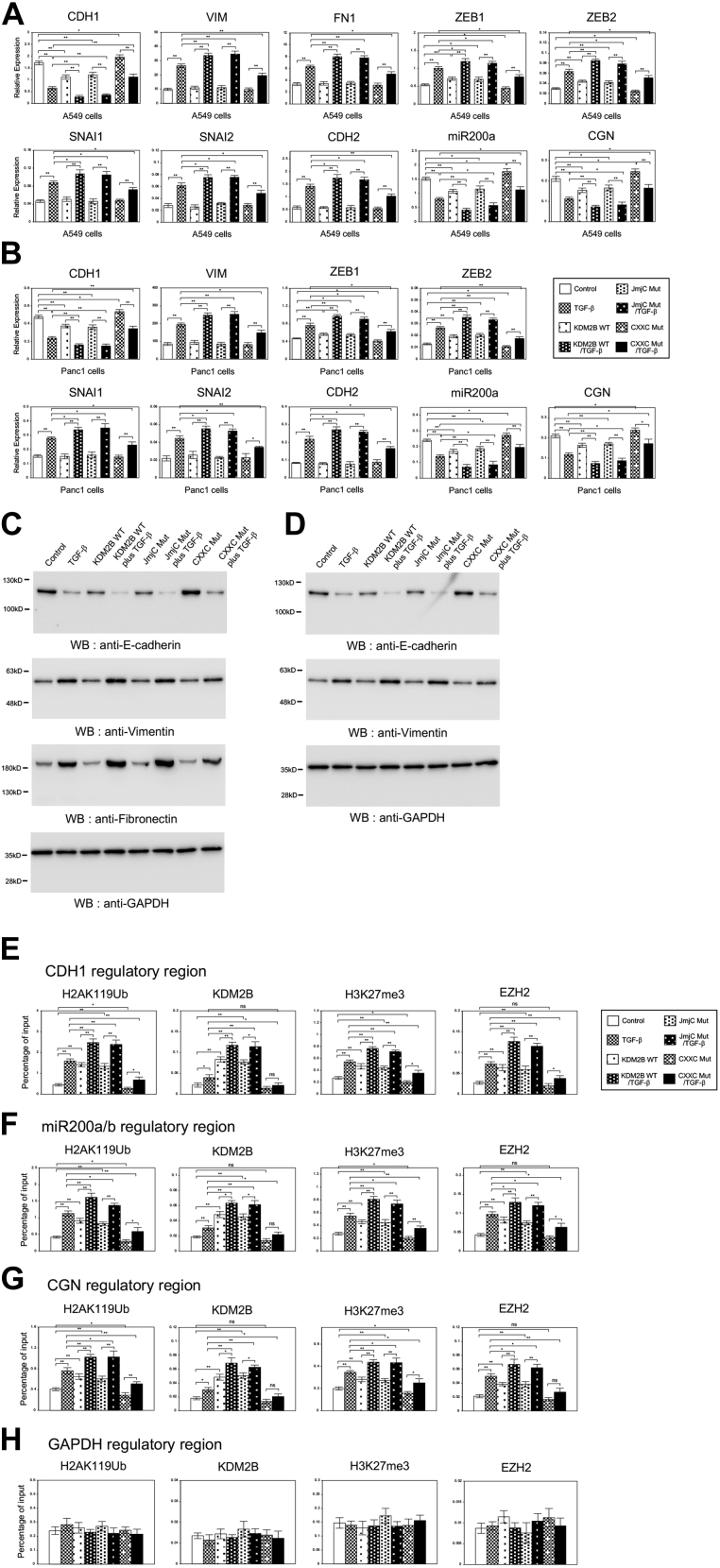
**Overexpression of *KDM2B* mutants showed different effects in the expression of several epithelial marker genes through the regulation of histone H2A ubiquitination and H3 methylation.***A*–*B*, QRT-PCR analysis of *CDH1*, *VIM*, *FN1*, *ZEB1*, *ZEB2*, *SNAI1*, *SNAI2*, *CDH2*, *miR200a*, and *CGN* in A549 (*A*) and Panc1 (*B*) cells infected with the control retrovirus or the retrovirus expressing wild-type *KDM2B* (WT), JmjC domain mutant (JmjC Mut) or CXXC motif mutant (CXXC Mut) with or without TGF-β for 24 h (n = 3) (∗∗*p* < 0.01; ∗*p* < 0.05). *C*–*D*, immunoblotting of E-cadherin, Fibronectin, Vimentin, and GAPDH proteins in A549 (*C*) and Panc1 (*D*) cells with wild-type and mutant *KDM2B* overexpression. *E*–*H*, ChIP analyses of H2AK119Ub, KDM2B, H3K27me3, and EZH2 on the regulatory regions of *CDH1* (*E*), *miR200a/b* (*F*), *CGN* (*G*), and *GAPDH* genes (*H*) in A549 cells with wild-type and mutant *KDM2B* overexpression (n = 3) (∗∗*p* < 0.01; ∗*p* < 0.05; ns, not significant).

To gain further insight into the epigenetic regulation of the genes by KDM2B, we constructed two types of KDM2B mutants for the overexpression experiments. The JmjC mutant is defective in histone H3 demethylase activity (30) and the CXXC mutant lacks DNA recognition property of KDM2B (31). A549 and Panc1 cells were infected with the control retrovirus or the retroviruses expressing wild-type KDM2B (WT) or the mutants, and the expression levels of them were confirmed by QRT-PCR (Fig. S8, *A*–*B*) and immunoblotting (Fig. S8, *C*–*D*). Then we analyzed the expression of EMT marker genes in A549 and Panc1 cells. In the case of epithelial genes such *CDH1*, *miR200a*, and *CGN*, JmjC mutant by itself decreased the expression similarly with wild-type KDM2B, but CXXC mutant increased the expression significantly in A549 and Panc1 cells (Fig. 5, *A*–*B*). JmjC mutant also enhanced the effect of TGF-β in the expression of these genes while CXXC mutant reduced it (Fig. 5, *A*–*B*). For *ZEB* family, JmjC mutant itself increased the expression similarly with wild-type, but CXXC mutant decreased the expression. Both mutants had no effect on other mesenchymal marker genes in the absence of TGF-β, but the effects of TGF-β were potentiated by JmjC mutant and counteracted by CXXC mutant (Fig. 5, *A*–*B*). Similar effects of these mutants were confirmed in protein expression of E-cadherin, Vimentin, and Fibronectin in both cells (Fig. 5, *C*–*D*). These results indicated that DNA recognition property but not histone H3 demethylase activity of KDM2B was indispensable for the downregulation of epithelial genes such as *CDH1*, *miR200a*, and *CGN* during EMT. In addition, it was suggested that DNA recognition defective mutant (CXXC mutant) might behave like a dominant negative mutant in the transcriptional regulation.

Next we examined the state of histone H2A ubiquitination and H3 methylation on the regulatory regions of *CDH1*, *miR200a*, and *CGN* genes in A549 cells expressing KDM2B mutants. The JmjC mutant showed the same properties with wild-type KDM2B for its recruitment, H2AK119Ub increase, EZH2 recruitment, and H3K27me3 increase on these regulatory regions in the presence or absence of TGF-β (Fig. 5, *E*–*G*). In contrast, CXXC mutant significantly reduced H2AK119Ub and H3K27me3 modification on these epithelial genes with or without TGF-β (Fig. 5, *E*–*G*). The decreases of KDM2B and EZH2 recruitment on these regulatory regions in the absence of TGF-β were not statistically significant, but TGF-β-dependent increases of their recruitment were significantly reduced by the overexpression of CXXC mutant (Fig. 5, *E*–*G*). We did not detect any changes in H2AK119Ub, KDM2B, H3K27me3, and EZH2 signals by KDM2B mutants on the regulatory region of unrelated *GAPDH* gene (Fig. 5*H*). These results indicated that DNA recognition property but not histone H3 demethylase activity of KDM2B was indispensable for its function regulating histone H2AK119Ub and H3K27me3 modification on the specific target genes including *CDH1*, *miR200a/b*, and *CGN*. Furthermore, the results of CXXC mutant suggested that this DNA recognition defective mutant of KDM2B might partially inhibit the function of endogenous PRC1 complex for transcriptional regulation. These results together suggested that DNA recognition property of KDM2B might play an important role in the selection of target genes for regulating histone H2AK119 ubiquitination and thereby affecting the subsequent EZH2 recruitment and H3K27me3 modification.

We further tried to examine whether KDM2B histone demethylase activity would be involved in the gene expression changes during EMT. Since KDM2B is a histone H3K36 demethylase (32), we first analyzed the effects of *KDM2B* knockdown or *KDM2B* overexpression on the overall levels of histone H3K36 methylation in A549 and Panc1 cells. We also examined the levels of H3K27 methylation because our results indicated KDM2B changed H3K27me3 levels at the local regulatory regions of several epithelial genes. The immunoblotting revealed that *KDM2B* knockdown did not change the overall levels of H3K36me2 and H3K27me3 in A549 and Panc1 cells (Fig. S9). This might be due to the compensatory effect of redundant function of other H3K36 demethylases. On the other hand, *KDM2B* overexpression decreased the overall levels of H3K36me2 significantly, but had no effect on H3K27me3 (Fig. S10), which was consistent with the results of the previous paper (32). These results suggested that KDM2B-mediated changes of H3K27 methylation were only observed at the specific target genes and possibly caused by KDM2B function as a component of PRC1 complex. Next we examined the state of H3K36 methylation at the local level on the regulatory regions of the epithelial genes such as *CDH1*, *miR200a*, and *CGN* and unrelated *GAPDH* gene in A549 cells. The ChIP analysis revealed that the levels of H3K36me2 were originally very low on the regulatory regions of the epithelial genes and not changed significantly by *KDM2B* knockdown or *KDM2B* overexpression (Figs. S11 and S12). In contrast, we observed that the levels of H3K4me3 on these regulatory regions were dynamically changed with *KDM2B* knockdown or *KDM2B* overexpression, which were proportional to the expression levels of these genes as reported previously (Figs. S11 and S12) (15, 16). There were no significant changes observed at the regulatory region of unrelated *GAPDH* gene (Figs. S11*D* and S12*D*). These results suggested that H3K36 methylation was not responsible for the transcriptional regulation of the epithelial genes including *CDH1*, *miR200a*, and *CGN* by KDM2B.

### Knockdown of EED, a core component of PRC2, inhibited KDM2B-mediated expression changes of EMT-related genes in EMT process

**Figure 6:**
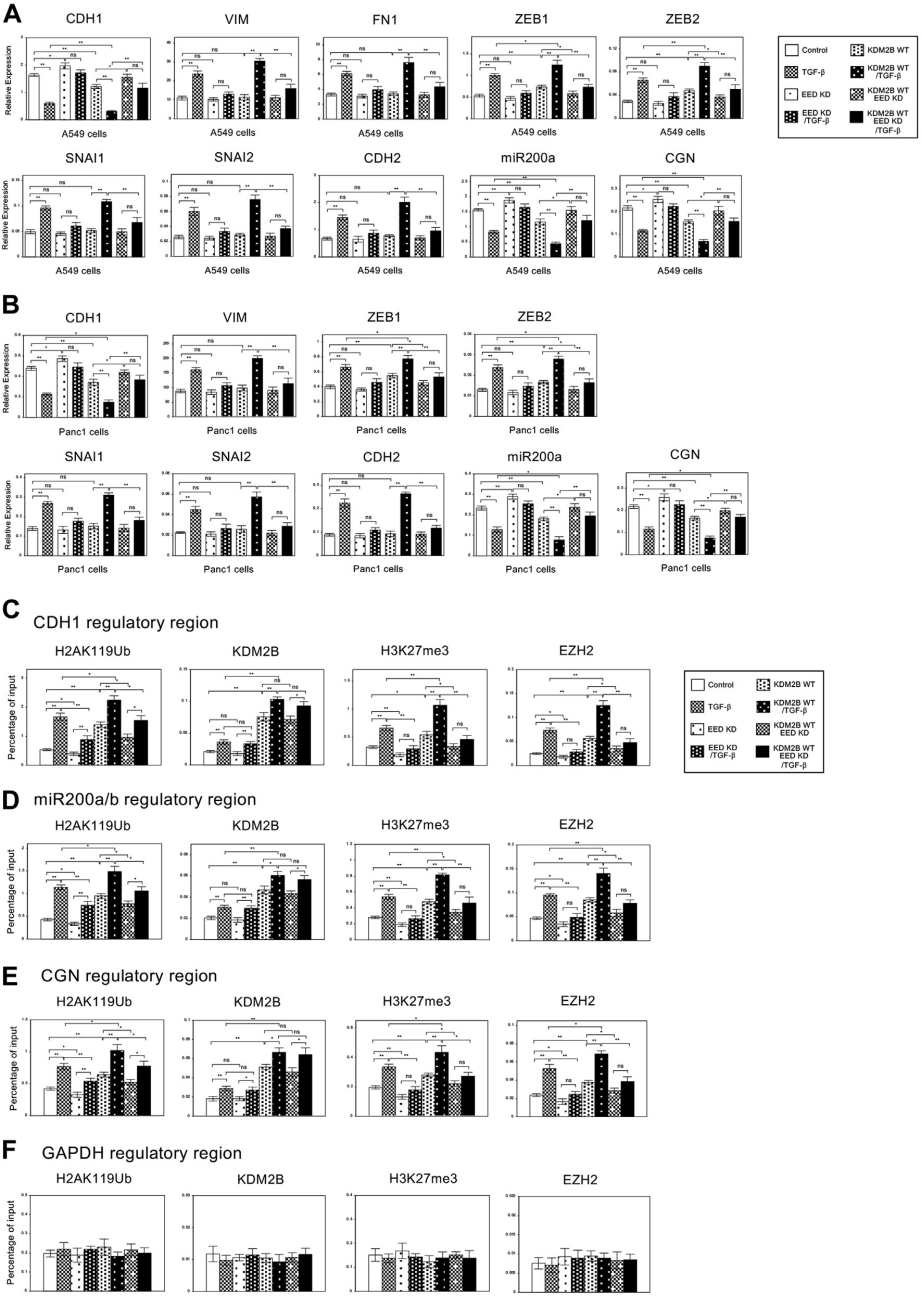
**Knockdown of *EED*, one of the core components of PRC2, showed inhibitory effects on TGF-β- or KDM2B- dependent expression changes of EMT-related marker genes during EMT.***A*–*B*, QRT-PCR analysis of *CDH1*, *VIM*, *FN1*, *ZEB1*, *ZEB2*, *SNAI1*, *SNAI2*, *CDH2*, *miR200a*, and *CGN* in the control or *EED* knockdown (EED shRNA#1) A549 (*A*) and Panc1 (*B*) cells coinfected with the control retrovirus or the retrovirus expressing wild-type *KDM2B* (WT) with or without TGF-β for 24 h (n = 3) (∗∗*p* < 0.01; ∗*p* < 0.05; ns, not significant). *C*–*F*, ChIP analyses of H2AK119Ub, KDM2B, H3K27me3, and EZH2 on the regulatory region of *CDH1* (*C*), *miR200a/b* (*D*), *CGN* (*E*), and *GAPDH* genes (*F*) in the control or *EED* knockdown A549 cells with wild-type *KDM2B* overexpression (n = 3) (∗∗*p* < 0.01; ∗*p* < 0.05; ns, not significant).

To clarify the relationship of KDM2B-containing PRC1 complex and PRC2 complex in the regulation of EMT-related genes, we examined the knockdown effects of EED, one of the core components of PRC2, in EMT process of A549 and Panc1 cells. We showed the data using *EED* shRNA#1 as a representative result because it caused efficient decrease of *EED* expression in the cells as reported previously (15). *EED* knockdown significantly increased the expression of epithelial marker genes such as *CDH1*, *miR200a*, and *CGN* and inhibited its transcriptional repression mediated by TGF-β in A549 and Panc1 cells (Fig. 6, *A*–*B*). For the mesenchymal markers, *EED* knockdown by itself had no influence on their expressions and prevented TGF-β-dependent increase of them as described previously (Fig. 6, *A*–*B*) (15). In the KDM2B-overexpressing cells, *EED* knockdown canceled KDM2B-induced downregulation of these epithelial genes, KDM2B-mediated upregulation of *ZEB* family, and TGF-β-dependent expression changes of all EMT-related genes (Fig. 6, *A*–*B*). These results indicated that EED was indispensable for KDM2B-dependent transcriptional regulation of EMT-related genes during EMT.

Next we examined how EED affected the KDM2B-mediated expression of epithelial genes such as *CDH1*, *miR200a*, and *CGN* in A549 cells by ChIP experiment (Fig. 6, *C*–*E*). As reported previously, *EED* knockdown by itself caused significant decrease of EZH2 recruitment and H3K27 methylation on these regulatory regions and antagonized TGF-β-effect on the increase of them (15). Importantly, *KDM2B*-dependent increase of EZH2 recruitment and H3K27 methylation was inhibited by *EED* knockdown (Fig. 6, *C*–*E*). These results indicated that EED was indispensable for *KDM2B*-mediated increase of EZH2 recruitment and H3K27 methylation on the regulatory regions of *CDH1*, *miR200a*, and *CGN* genes. For KDM2B recruitment, *EED* knockdown had no influence on the effects of TGF-β treatment and KDM2B overexpression (Fig. 6, *C*–*E*). *EED* knockdown could not cancel the effects of TGF-β or *KDM2B* overexpression on the increase of H2AK119Ub, but showed significant inhibition on its increase (Fig. 6, *C*–*E*). These results suggested that KDM2B-independent mechanism for H2A ubiquitination might be partly involved in this process. These observed changes were specific to the regulatory regions of *CDH1*, *miR200a*, and *CGN* genes and not detected at the regulatory region of unrelated *GAPDH* gene (Fig. 6*F*). The *EED* knockdown studies suggested that PRC2 function indicated by EZH2 recruitment and H3K27 methylation was indispensable for KDM2B-mediated transcriptional repression of *CDH1*, *miR200a*, and *CGN* genes. Together with the ChIP results shown in Figures 3 and 4, it was concluded that KDM2B-mediated H2A ubiquitination resulted in the subsequent increase of EZH2 binding and H3K27me3 levels on the regulatory regions and that these PRC1 and PRC2 activities were both indispensable for the transcriptional repression of the epithelial genes during EMT process.

## Discussion

In this study, we found that KDM2B was indispensable for TGF-β-induced EMT of A549 lung cancer and Panc1 pancreatic cancer cell lines. *KDM2B* knockdown inhibited EMT phenotypes by antagonizing TGF-β-dependent changes in the expression of EMT-related genes. Overexpression of *KDM2B* and its mutants revealed that KDM2B was involved in transcriptional repression of several epithelial marker genes such as *CDH1*, *miR200a*, and *CGN* through its function as a variant PRC1 component. From the mechanistic investigations, it was strongly suggested that KDM2B could specifically recognize the regulatory regions of these epithelial genes and mediate histone H2AK119 monoubiquitination, thereby activating the EZH2 recruitment and histone H3K27 methylation for gene repression. This study revealed a novel functional significance of KDM2B in the epigenetic control of EMT process of lung and pancreatic cancer cells.

The polycomb group (PcG) proteins constitute PRC1 and PRC2 complex that function as epigenetic regulators of gene expression in cell fate, development, and cancer (20, 21). While PRC2 induces histone H3K27 methylation by its catalytic subunit EZH2, PRC1 monoubiquitylates H2AK119 through its catalytic subunit, RING1A or RING1B ubiquitin ligase, for transcriptional repression. A growing body of evidence indicated that increased activity of PRC2 or EZH2 was responsible for malignant phenotypes of cancer cells (23, 33). Especially, PRC2 was shown to be involved in the critical step during EMT. It has been reported that EZH2 downregulated *CDH1/E-cadherin* expression by inducing histone H3K27 methylation on its promoter region (6). In addition, we found that EED, a core component of PRC2, and JARID2, an accessary factor of PRC2, were essential for TGF-β-induced EMT through *CDH1* repression in cancer cells (15, 16). On the other hand, several studies have demonstrated the role of PRC1 members in EMT process. For example, it was shown that BMI1, a component of PRC1, acted cooperatively with TWIST1, one of EMT-related transcription factors (EMT-TFs), to repress *CDH1* during EMT in head and neck squamous cell carcinoma (34). RING1A/1B was reported to interact with SNAI1, another EMT-TF, and contribute to SNAI1-mediated repression of *CDH1* in pancreatic cancer cells (35). In this report, we discovered that KDM2B, a member of variant PRC1 complex and also a histone H3 demethylase, epigenetically regulated the downregulation of several epithelial marker genes including *CDH1*, which was indispensable for TGF-β-induced EMT of lung and pancreatic cancer cells. Since the JmjC mutant of KDM2B exhibited the similar properties with wild-type KDM2B in the regulation of EMT-related genes, histone H3 demethylase activity of KDM2B was suggested to be dispensable for its function in EMT. The ChIP experiments (Figs. S11 and S12) also revealed that H3K36 methylation was not responsible for the transcriptional regulation of these epithelial genes by KDM2B, although KDM2B is a histone H3K36 demethylase. Therefore, our studies validated the importance of KDM2B function that regulates the PRC1 activity in EMT-inducing transcriptional program.

**Figure 7:**
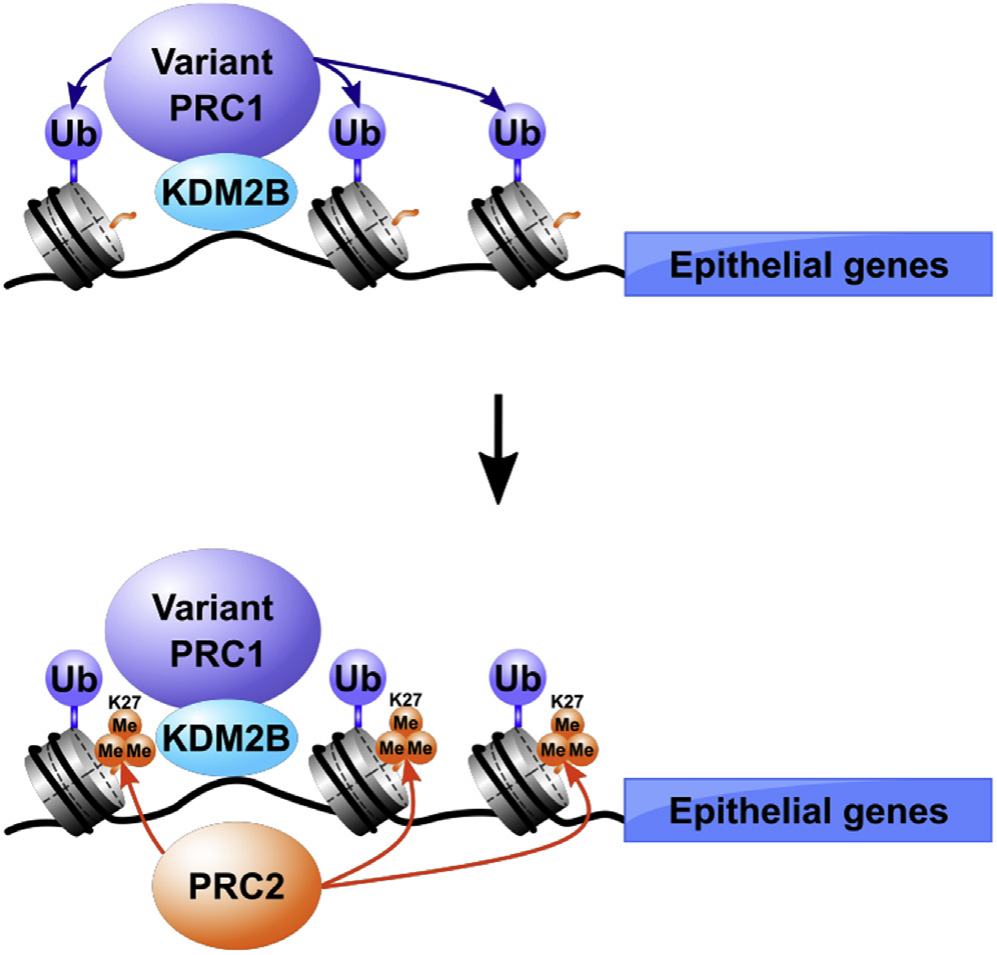
**Schematic representation of the possible mechanism for KDM2B-mediated epigenetic regulation of the epithelial genes such as *CDH1*, *miR200a*, and *CGN* during EMT**.

Currently, PRC1 complex is divided into two subfamilies of canonical PRC1 and variant PRC1. Both PRC1 exist in different forms with distinct subunit compositions influencing the mechanistic properties of the complexes (24). The canonical PRC1 complex can recognize H3K27me3 and is recruited in a hierarchical manner to the sites modified by PRC2 for gene repression (21). The above two papers reporting the involvement of BMI1 and RING1A/1B in EMT have shown that prior modification of H3K27 by PRC2 was important for TWIST- or SNAI1-mediated recruitment of canonical PRC1 containing BMI1 and/or RING1A/1B on the *CDH1* promoter. In contrast, our experiments showed that knockdown of *KDM2B* or *RING1A/1B* (Fig. 4) inhibited TGF-β-dependent increase of not only H2AK119Ub, but also H3K27me3 and EZH2 binding on the regulatory regions of the epithelial genes including *CDH1* for transcriptional repression. These results suggested that the H2AK119 ubiquitination on these regulatory regions by the variant PRC1 complex including KDM2B and RING1A/1B was necessary for the subsequent PRC2 activation indicated by the increase of EZH2 binding and H3K27me3 levels. This is consistent with the proposed model of variant PRC1 function in gene repression in the absence of pre-existing H3K27me3 reported in the previous papers (See the possible mechanism in Fig. 7) (26, 27). The variant PRC1 complex can recognize specific target genes to induce H2A ubiquitination, and then PRC2 is recruited to the sites in a hierarchical manner for H3K27 methylation. Furthermore, knockdown experiments of EED, a core component of PRC2, suggested that PRC2 function was indispensable for KDM2B-mediated transcriptional repression of *CDH1*, *miR200a*, and *CGN* genes. These results together indicated that PRC1 and PRC2 activities were both necessary for the transcriptional repression of these epithelial genes during EMT process. However, further studies will be required to address how PRC1 and PRC2 complexes interact with each other on the target genes to induce transcriptional repression.

In our experiments, DNA recognition defective mutant (CXXC) of KDM2B reduced TGF-β-dependent increases of endogenous KDM2B recruitment and H2AK119Ub modification on the regulatory regions of the epithelial genes including *CDH1*, *miR200a*, and *CGN* and might function as a dominant negative mutant in the transcriptional repression of these genes. This mutant also decreased the subsequent increases of EZH2 binding and H3K27 trimethylation on the genes. Taken together, the DNA recognition property of KDM2B was indispensable for its function to recognize the specific target genes and regulate gene repression through PRC1 activity during EMT (Fig. 7). These results are consistent with the previous reports showing that KDM2B recognized the genomic DNA in CpG islands (CGIs) *via* its CXXC domain and contributed to recruit variant PRC1 complex for gene repression independently of pre-existing H3K27 methylation in mouse embryonic stem (ES) cells (36, 37). The CGIs were found in the regulatory regions of *CDH1*, *miR200a*, and *CGN* genes and were included in the probes used in our ChIP experiments. These regulatory regions revealed KDM2B binding, H2AK119 ubiquitination, subsequent EZH2 recruitment, and H3K27 methylation in the ChIP assays, which was correlated well with the transcriptional repression. Therefore, it was considered that the recognition of CGIs by KDM2B played a crucial role in the determination of target specificity. However, in contrast to ES cells, the KDM2B-binding CGIs have not been extensively analyzed in cancer cells such as A549 and Panc1 cells. Further examinations are warranted to clarify how KDM2B selects the specific target genes in cancer cells.

Among the mesenchymal marker genes, *ZEB1* and *ZEB2* were downregulated by *KDM2B* knockdown and upregulated by *KDM2B* overexpression (Figs. 1 and 2). Since *ZEB* family is a target of *miR200a*, the expression levels of *ZEB* family were inversely correlated with the levels of *miR200a*. Therefore, it was suggested that KDM2B affected the expression of *ZEB1* and *ZEB2* indirectly through the epigenetic regulation of *miR200a*. This is also consistent with our results in the previous papers (15, 16). For other mesenchymal marker genes, *KDM2B* knockdown or *KDM2B* overexpression by itself had no effect in the expression of them but potentiated the effects of TGF-β (Figs. 1 and 2). We tried to examine the effects of KDM2B on the mesenchymal genes such as *FN1* and *VIM* by ChIP assays. We analyzed the state of H2AK119Ub, KDM2B recruitment, H3K27me3, EZH2 binding, H3K36me2, and H3K4me3 on the regulatory regions of *FN1* and *VIM* genes in A549 cells with *KDM2B* knockdown or *KDM2B* overexpression (Figs. S13 and S14). We did not detect any significant levels of signals in H2AK119Ub, KDM2B, H3K27me3, EZH2, and H3K36me2 to see the effects of KDM2B. These results suggested that KDM2B was not directly involved in the epigenetic regulation of *FN1* and *VIM* genes. We could observe that the levels of H3K4me3 on these regulatory regions were dynamically changed with TGF-β treatment, *KDM2B* knockdown, or *KDM2B* overexpression (Figs. S13 and S14). These changes of H3K4me3 appeared to be proportional to the expression levels of these genes. In mammalian cells, H3K27 methylation is catalyzed by polycomb (PcG) family and H3K4 methylation is mostly regulated by COMPASS family proteins (38). These two families are well known for their opposing roles in balancing gene expression. In this study, we mainly focused on the function of PcG family, PRC1 and PRC2, in transcriptional repression during EMT. However, it should be the next important subject to investigate the regulation of H3K4 methylation in EMT process.

A previous report showed that KDM2B knockdown and overexpression influenced the expression of both epithelial and mesenchymal genes in HCT116 colon cancer cell line (39), indicating a relationship between KDM2B expression and EMT. However, in colon cancer cells, it remains unclear whether KDM2B-induced EMT was dependent on its histone demethylase activity or PRC1 activity. Furthermore, a lot of evidences have revealed that histone modifying enzymes such as KDM2B are involved in the initiation and progression of human cancer (11, 12). These epigenetic regulatory factors have been sometimes reported to act as oncogene products in some types of cancers or to reveal tumor suppressive function in other cancers, suggesting that their contribution to cancer is highly dependent on many factors and cellular contexts (40). Therefore, careful analyses and discussions are critical to understand their roles in different types of cancers. In this paper, we have found an essential function of KDM2B as a component of variant PRC1 complex during TGF-β-induced EMT of A549 lung cancer and Panc1 pancreatic cancer cell lines. KDM2B played a crucial role in EMT process by regulating the expression of several epithelial marker genes such as *CDH1*, *miR200a*, and *CGN* through the modulation of histone H2A ubiquitination. Our study provides novel insights into the involvement of variant PRC1 complex for epigenetic mechanism of cancer malignancies.

## Experimental procedures

### Plasmids, cell culture, transfections, and antibody

For the knockdown experiments, lentiviral vectors expressing small hairpin RNAs (shRNAs) were constructed as described previously (41). The pLKO.1-Puro plasmid (Sigma-Aldrich, St Louis, USA) and its derivative, pLKO.1-Neo plasmid were used for the combined knockdown. The oligonucleotide sequences for shRNAs were described previously (16) and are listed in Table S1. We usually used two different shRNAs (shRNA#1 and shRNA#2) for each gene. Two shRNAs have similar efficiencies for knockdown of each gene (Figs. S3, *A*–*B* and S5) and have similar effects on EMT phenotypes judged from the marker gene expression (Fig. S3, *C*–*D*). Thus, the data of one representative shRNA for each gene were shown in the main figures and the text. For cloning of human *KDM2B* cDNA, the primer sets described in Table S1 were designed based on the reference sequence (ENST00000636052.1) in GENCODE database. The two amplified cDNAs were connected with HincII site and cloned into pDON-5 Neo plasmid (Takara, Ohtsu, Japan) to produce retrovirus expressing KDM2B. *KDM2B* JmjC mutant (H242A) that is catalytically deficient in histone H3 demethylase activity (30) was constructed by PrimeSTAR Mutagenesis Basal kit (Takara, Ohtsu, Japan) using JmjC mutant primer set described in Table S1. *KDM2B* CXXC mutant (C613A, C616A, C619A) abolishing the DNA recognition property (31) was also constructed by the same kit. The C613A mutation was introduced to wild-type *KDM2B* using CXXC mutant1 primer set (Table S1) and then the C616A and C619A mutations were simultaneously introduced with CXXC mutant2 primer set (Table S1). The nucleotide exchanges of the mutants were confirmed by DNA sequencing (Fig. S15). The sequence-verified PCR products were used for cloning and plasmid construction. Human lung cancer and pancreatic cancer cell lines, A549 and Panc1, were obtained from ATCC and maintained in Dulbecco’s modified Eagle’s medium (DMEM) with 10% FBS, 2 mM glutamine, and penicillin/streptomycin (Sigma-Aldrich, St Louis, USA) at 37 °C in 5% CO2. A549 and Panc1 cells are a good model system for EMT showing rapid and clear changes for cell morphology and EMT-related gene expression induced by treatment of 1 ng/ml of TGF-β (R&D Systems, Minneapolis, USA) for 24 to 72 h. The methods for the production and infection of shRNA-expressing lentiviruses or cDNA-expressing retroviruses were essentially the same as described previously (8). For the preparation of anti-KDM2B rabbit polyclonal antibody, histidine-tagged N-terminal KDM2B (M1 to Y396) protein purified from genetically engineered *Escherichia coli* was used.

### Single-guide RNAs (sgRNAs) and CRISPR/Cas9 screening

The oligonucleotides for five different sgRNAs against 23 PRC1-related genes were designed with CRISPRdirect (http://crispr.dbcls.jp) and listed in Table S2. Linker’s sequences (5’-cacc-3’ for forward primer and 5’-aaac-3’ for reverse primer) were attached to the synthesized oligos, and the annealed oligonucleotides were cloned into the modified pLX-sgRNA plasmid (Addgene plasmid #50662) digested with BsmBI (New England Biolabs, Ipswich, USA). The modified plasmid termed pLX-sgRNA ver.2 (Fig. S1*A*) was constructed by combining original pLX-sgRNA (42) and a part of lentiGuide-Puro plasmid (Addgene plasmid #52963) (43) (See the detailed procedure described in Method S1). The methods for the production and infection of sgRNA- or Cas9-expressing lentiviruses were essentially the same as described previously (8). Doxycycline-inducible Cas9-expressing A549 cells were established by the infection of pCW-Cas9 (Addgene plasmid #50661)-derived virus (42), and the cell clone (A549/Cas9h) with the highest Cas9 induction was isolated (Fig. S1*B*). The A549/Cas9h cells were infected with lentiviruses expressing five sgRNAs for each gene and treated with 10 μg/ml doxycycline (Wako, Osaka, Japan) for 48 h and then used for the screening experiments (Fig. S1*C*). To confirm the knockdown efficiencies by the sgRNAs, quantitative RT-PCR (QRT-PCR) was performed as described below (Fig. S2*A*). Primers used for QRT-PCR are listed in Table S3.

### Quantitative PCR

Total RNA was extracted with RNAiso plus (Takara, Ohtsu, Japan) using a standard method and transcribed to cDNA using SuperScript Vilo cDNA synthesis kit (Invitrogen, Waltham, USA). Quantitative RT-PCR (QRT-PCR) was performed as described previously (8). PCR data were normalized with control human *GAPDH* expression. The averages from at least three independent experiments are shown with the standard deviations. *p*-values were calculated between control and the samples using Student’s *t*-test. Primers used for the quantitative PCR were described previously (8, 16, 41) and are listed in Tables S1 and S3.

### Cell staining, immunofluorescence, immunoprecipitation, and immunoblotting

To observe cell morphologies, A549 or Panc1 cells were fixed in 4% paraformaldehyde and stained with 0.4% crystal violet. To visualize actin cytoskeleton, the cells were stained with 0.25 μM tetramethylrhodamine isothiocyanate (TRITC)-conjugated phalloidin (Sigma, St Louis, USA). For indirect immunofluorescence, the specimens were treated with anti-E-cadherin antibody (#610181, BD Biosciences, San Jose, USA) and incubated with Alexa546-conjugated anti-mouse IgG antibody (Invitrogen, Waltham, USA). Nuclei were stained with 4’,6-diamidino-2-phenylindole (DAPI). For immunoprecipitation and immunoblotting, cells were lysed in RIPA buffer as described previously (19). The lysates were immunoprecipitated with anti-KDM2B antibody coupled with Protein G sepharose 4 fast flow (GE Healthcare, South East England, UK). The precipitates or lysates were separated on SuperSep Ace 10% running gel (Wako, Osaka, Japan) and transferred to Hybond-LFP membrane (GE Healthcare, USA). The antibodies that were used in this study include anti-E-cadherin, anti-Vimentin (ab8069, Abcam, Cambridge, USA), anti-Fibronectin (SAB4500974, Sigma, St Louis, USA), antiphosphorylated SMAD3 (ab51451, Abcam, Cambridge, USA), anti-GAPDH (6C5, Millipore, Billerica, USA), anti-FLAG (F1804, Sigma, St Louis, USA), anti-RING1A (D2P4D, Cell Signaling Tech, Danvers, USA), anti-RING1B (D22F2, Cell Signaling Tech, Danvers, USA), anti-PHC1 (1F3F3, Cell Signaling Tech, Danvers, USA), anti-SKP1 (D3J4N, Cell Signaling Tech, Danvers, USA), anti-RYBP antibodies (D8J7W, Cell Signaling Tech, Danvers, USA), and anti-TBP (22006-1-AP, Proteintech, Rosemont, USA).

### Cell migration and invasion assays

Cell migration and invasion assays were carried out by using modified Boyden chambers consisting of Transwell membrane filter inserts (#3422, Corning, Corning, USA) as described previously (17). For cell invasion, the upper surfaces of the membranes were coated with 1 mg/ml Matrigel matrix (BD Bioscience, USA). Briefly, serum-starved cells (2 × 10^5^) were suspended in the upper chamber in DMEM containing 1 mg/ml BSA and 0.5% FBS. The lower chamber was filled with DMEM containing 10% FBS. The chambers were incubated for 24 h (A549) and 36 h (Panc1) for cell migration, 36 h (A549) and 48 h (Panc1) for cell invasion. For TGF-β-treated cells, cells were pretreated with 1 ng/ml of TGF-β for 4 days and then allowed to migrate. Cells remaining on the upper surface of the membrane were removed by wiping with a cotton bud, and cells on the underside of the filter were fixed with 4% paraformaldehyde and stained with 0.4% crystal violet. Cells were counted under a microscope in at least eight different fields and two independent experiments.

### Chromatin immunoprecipitation (ChIP) assays

ChIP assays were carried out essentially the same as described previously (41, 44). In brief, cells were cross-linked with 1% paraformaldehyde for 10 min and chromatins were fragmented by Bioruptor II ultrasonicator (BM Equipment Co, Tokyo, Japan). The cross-linked cell lysates were immunoprecipitated with the antibodies (anti-H2AK119Ub (#8240, Cell Signaling Tech., Danvers, USA), anti-KDM2B, anti-H3K27me3 (44), anti-H3K36me2 (44), anti-H3K4me3 (44), and anti-EZH2 (#17-662, Millipore, Billerica, USA)). Quantitative PCR was performed to detect the enrichment of specific amplified region. Percentage enrichment over input chromatin was presented. Primers used for the QPCR corresponding to the regulatory region h of *CDH1* gene, region a of *miR200a/b* gene, and region a of *GAPDH* gene are described previously (8). Other primers for the regulatory regions were listed in Table S1.

## Data availability

All data are included within the article and supporting information. The materials and methods in this study are available from the corresponding author upon reasonable request.

## Conflict of interest

The authors declare that they have no potential conflicts of interest with the contents of this article.
