# Supplementary material for: KDM2B is involved in the epigenetic regulation of TGF-β-induced epithelial–mesenchymal transition in lung and pancreatic cancer cell lines
Source: J Biol Chem. 2020 Dec 24;296:100213. doi: 10.1074/jbc.RA120.015502 (PMC7948487; doi:10.1074/jbc.RA120.015502)
Supplement: Supplemental Figures and Tables [file mmc1.docx]

**Supporting information (SI)**

**Wanna-udom S. et al.**

1. **Supplementary Table S1**
2. **Supplementary Table S2**
3. **Supplementary Table S3**
4. **Supplementary Method S1**

**4. Supplementary Figure S1**

**5. Supplementary Figure S2**

**6. Supplementary Figure S3**

**7. Supplementary Figure S4**

**8. Supplementary Figure S5**

**9. Supplementary Figure S6**

**10. Supplementary Figure S7**

**11. Supplementary Figure S8**

**12. Supplementary Figure S9**

**13. Supplementary Figure S10**

**14. Supplementary Figure S11**

**15. Supplementary Figure S12**

**16. Supplementary Figure S13**

**17. Supplementary Figure S14**

**17. Supplementary Figure S15**

**Supplementary Table S1**

The sequences of oligonucleotides for shRNA-expression, quantitative PCR and mutant construction used in the main text of this paper. The mutation sites were underlined.

| **Name** | **Primer sequence (5’ to 3’ )** |
| --- | --- |
| *KDM2B shRNA#1* | F: CCGGGATGAGCGTGAAAGGTTGTTTCTCGAGAAACAACCTTTCACGCTCATCTTTTTG  R: AATTCAAAAAGATGAGCGTGAAAGGTTGTTTCTCGAGAAACAACCTTTCACGCTCATC |
| *KDM2B shRNA#2* | F: CCGGCTGAACCACTGCAAGTCTATCCTCGAGGATAGACTTGCAGTGGTTCAGTTTTTG  R: AATTCAAAAACTGAACCACTGCAAGTCTATCCTCGAGGATAGACTTGCAGTGGTTCAG |
| *RING1A shRNA#1* | F: CCGGGCCCTGATCTCTAAGATCTATCTCGAGATAGATCTTAGAGATCAGGGCTTTTT  R: AATTAAAAAGCCCTGATCTCTAAGATCTATCTCGAGATAGATCTTAGAGATCAGGGC |
| *RING1A shRNA#2* | F: CCGGGATTCTTCTATATTGTACAGACTCGAGTCTGTACAATATAGAAGAATCTTTTTTG  R: AATTCAAAAAAGATTCTTCTATATTGTACAGACTCGAGTCTGTACAATATAGAAGAATC |
| *RING1B shRNA#1* | F: CCGGCGAAGTCTACACAGTGAATTACTCGAGTAATTCACTGTGTAGACTTCGTTTTTG  R: AATTCAAAAACGAAGTCTACACAGTGAATTACTCGAGTAATTCACTGTGTAGACTTCG |
| *RING1B shRNA#2* | F: CCGGGCCAGGATCAACAAGCACAATCTCGAGATTGTGCTTGTTGATCCTGGCTTTTTG  R: AATTCAAAAAGCCAGGATCAACAAGCACAATCTCGAGATTGTGCTTGTTGATCCTGGC |
| *KDM2B*  *QPCR* | F: GAGGAGAAGAAGAAGGTGAAG  R: TTGATGGGCTGCTGGTTC |
| *RING1A*  *QPCR* | F: CTGCCAGACGAGGTATGTGA  R: CCCTGCTTCCTGCTGTTG |
| *RING1B*  *QPCR* | F: GATGACAGTGCACAGACGAGA  R: TCACCTTTGCTTCGAAGTTCT |
| *CGN*  *QPCR* | F: TGAGCTTACCCGAAAAGTGG  R: TTGGGATGGCTCTAGCTTCT |
| *KDM2B*  *cDNA1* | F: CATCTTTGAGTGCCGGGAGT  R: GCGTGGTTACACTTCGGACA |
| *KDM2B*  *cDNA2* | F: GCAATGAAATCATCCACCCTG  R: TGTTGTCCCACGTTCCAAATG |
| *KDM2B*  *JmjC mutant* | F: GACTTCGCCATCGACTTTGGAGGCACT  R: GTCGATGGCGAAGTCGGTGAAACAACC |
| *CXXC mutant1* | F: ACGCGAGCCCGCAAGTGCGAGGCCTGC  R: CTTGCGGGCTCGCGTCCGGCGCCGCCG |
| *CXXC mutant2* | F: AGGCCGAGGCCGCCCTGCGGACCGAGT  R: GGGCGGCCTCGGCCTTGCGGGCTCGCG |
| *CGN ChIP3* | F: GCAGTTGCGAATCAGAGACA  R: CACGAAAGCAAAGGACACAA |
| *FN1 ChIP2* | F: AGTTGCCACCAAGTTTGCTTCC  R: CCACCTTCTTGGAGGCGACA |
| *VIM ChIP3* | F: GGCCCAGCTGTAAGTTGGTA  R: CCTAGCGGTTTAGGGGAAAC |

F: Forward, R: Reverse

**Supplementary Table S2**

A list of oligonucleotide sequences for PRC1-specific sgRNAs in this study

| **Target gene** |  | **5’- CACC- forward primer** | **5’-AAAC- reverse primer** |
| --- | --- | --- | --- |
| **1. PCGF1** | **#1** | CACCGCCAGATTGCGATCGCGATG | AAACCATCGCGATCGCAATCTGGC |
|  | **#2** | CACCGCCTCATCGCGATCGCAATC | AAACGATTGCGATCGCGATGAGGC |
|  | **#3** | CACCGATGGACCCGCTACGGAACG | AAACCGTTCCGTAGCGGGTCCATC |
|  | **#4** | CACCGCACGAAGTAGCCGGCGCAT | AAACATGCGCCGGCTACTTCGTGC |
|  | **#5** | CACCGCAGGACATCGTGTATAAGC | AAACGCTTATACACGATGTCCTGC |
| **2. PCGF2** | **#1** | CACCGATCGACGCCACCACTATCG | AAACCGATAGTGGTGGCGTCGATC |
|  | **#2** | CACCGACCTGCATCGTGCGCTACC | AAACGGTAGCGCACGATGCAGGTC |
|  | **#3** | CACCGGCACCTTCGTAGAATTCGA | AAACTCGAATTCTACGAAGGTGCC |
|  | **#4** | CACCGGATGAAGTACCCCCCGCAG | AAACCTGCGGGGGGTACTTCATCC |
|  | **#5** | CACCGAATCATGCATCGGACTACA | AAACTGTAGTCCGATGCATGATTC |
| **3. PCGF3** | **#1** | CACCGGATGAGGTACCCGCTGCAC | AAACGTGCAGCGGGTACCTCATCC |
|  | **#2** | CACCGCACCGTGGTGGCGTCGATG | AAACCATCGACGCCACCACGGTGC |
|  | **#3** | CACCGTGCAGACACTCGGTCACCG | AAACCGGTGACCGAGTGTCTGCAC |
|  | **#4** | CACCGTTTGAACTGTCGTCTGCTT | AAACAAGCAGACGACAGTTCAAAC |
|  | **#5** | CACCGTACCACCGCAGCGACGAGC | AAACGCTCGTCGCTGCGGTGGTAC |
| **4. PCGF4** | **#1** | CACCGACAAAGCACACACATCAGG | AAACCCTGATGTGTGTGCTTTGTC |
|  | **#2** | CACCGTCCACAAAGCACACACATC | AAACGATGTGTGTGCTTTGTGGAC |
|  | **#3** | CACCGAACGTGTATTGTTCGTTACC | AAACGGTAACGAACAATACACGTTC |
|  | **#4** | CACCGCCTTATATTCAGTAGTGGTC | AAACGACCACTACTGAATATAAGGC |
|  | **#5** | CACCGCCAGACCACTACTGAATATA | AAACTATATTCAGTAGTGGTCTGGC |
| **5. PCGF5** | **#1** | CACCGGCCACGAATGGCTACCCAA | AAACTTGGGTAGCCATTCGTGGCC |
|  | **#2** | CACCGGCTACCCAAAGGAAACACT | AAACAGTGTTTCCTTTGGGTAGCC |
|  | **#3** | CACCGATCAAGCCAACAACAGTGA | AAACTCACTGTTGTTGGCTTGATC |
|  | **#4** | CACCGGGCATTCCGTCACTGTTGT | AAACACAACAGTGACGGAATGCCC |
|  | **#5** | CACCGGAACTTGGTTGCCACACCT | AAACAGGTGTGGCAACCAAGTTCC |
| **6. PCGF6** | **#1** | CACCGGTGTCTCTCCCGACCATGG | AAACCCATGGTCGGGAGAGACACC |
|  | **#2** | CACCGCTCGTCCTCGTCCTCGAAG | AAACCTTCGAGGACGAGGACGAGC |
|  | **#3** | CACCGGTAGGCGCTGCCAAAACCG | AAACCGGTTTTGGCAGCGCCTACC |
|  | **#4** | CACCGTAGGCGCTGCCAAAACCGA | AAACTCGGTTTTGGCAGCGCCTAC |
|  | **#5** | CACCGGCTGCAGCTCCCTCGGTTT | AAACAAACCGAGGGAGCTGCAGCC |
| **7. RING1A** | **#1** | CACCGCCGGTCACCATGACGACGC | AAACGCGTCGTCATGGTGACCGGC |
|  | **#2** | CACCGGCATTCGCCGGCGTCGTCA | AAACTGACGACGCCGGCGAATGCC |
|  | **#3** | CACCGCCTACCTGCCGAAAGAAGC | AAACGCTTCTTTCGGCAGGTAGGC |
|  | **#4** | CACCGGTGTGAGGCGGCCGATACC | AAACGGTATCGGCCGCCTCACACC |
|  | **#5** | CACCGAAGTTGGGGTCTGGCCGTA | AAACTACGGCCAGACCCCAACTTC |
| **8. RING1B** | **#1** | CACCGGAGTTACAACGAACACCTC | AAACGAGGTGTTCGTTGTAACTCC |
|  | **#2** | CACCGAATTCACTGTGTAGACTTCG | AAACCGAAGTCTACACAGTGAATTC |
|  | **#3** | CACCGACAAAGAATGTCCTACCTGT | AAACACAGGTAGGACATTCTTTGTC |
|  | **#4** | CACCGTCTGGCCTTAGTGATCTTT | AAACAAAGATCACTAAGGCCAGAC |
|  | **#5** | CACCGCACTAGCACCATCCATTAC | AAACGTAATGGATGGTGCTAGTGC |
| **9. CBX2** | **#1** | CACCGCCAGCCGCGCCACTTGACC | AAACGGTCAAGTGGCGCGGCTGGC |
|  | **#2** | CACCGAGTACCTGGTCAAGTGGCG | AAACCGCCACTTGACCAGGTACTC |
|  | **#3** | CACCGGTGCTGAAGACCGCCCGGA | AAACTCCGGGCGGTCTTCAGCACC |
|  | **#4** | CACCGCTGAGCAGCGTGGGCGAGC | AAACGCTCGCCCACGCTGCTCAGC |
|  | **#5** | CACCGAGGTGCAGAACCGGAAGAG | AAACCTCTTCCGGTTCTGCACCTC |
| **10. CBX4** | **#1** | CACCGCCTGCACCACTAGCGGTTT | AAACAAACCGCTAGTGGTGCAGGC |
|  | **#2** | CACCGACATTGGAACGACGGGCAA | AAACTTGCCCGTCGTTCCAATGTC |
|  | **#3** | CACCGGGCGAGCACGTCTTCGCGG | AAACCCGCGAAGACGTGCTCGCCC |
|  | **#4** | CACCGGAGAAGAAGCGGATCCGCA | AAACTGCGGATCCGCTTCTTCTCC |
|  | **#5** | CACCGTATAACACGTGGGAACCGG | AAACCCGGTTCCCACGTGTTATAC |
| **11. CBX6** | **#1** | CACCGATCAAACGGCGGATCCGAA | AAACTTCGGATCCGCCGTTTGATC |
|  | **#2** | CACCGGAGGGAGCGTGAGCTGTAT | AAACATACAGCTCACGCTCCCTCC |
|  | **#3** | CACCGGGGACGGCGGGACATACGG | AAACCCGTATGTCCCGCCGTCCCC |
|  | **#4** | CACCGTGGGTGCCGCTGAGCAAGA | AAACTCTTGCTCAGCGGCACCCAC |
|  | **#5** | CACCGCGAATCCATCATCAAACGG | AAACCCGTTTGATGATGGATTCGC |
| **12. CBX7** | **#1** | CACCGCTGTCAGCCATCGGCGAGC | AAACGCTCGCCGATGGCTGACAGC |
|  | **#2** | CACCGGCGAACACCTGCTCGCCGA | AAACTCGGCGAGCAGGTGTTCGCC |
|  | **#3** | CACCGCAGGAAGAGCTCCCGTCGA | AAACTCGACGGGAGCTCTTCCTGC |
|  | **#4** | CACCGCGTAGGCCATGACGAGGCG | AAACCGCCTCGTCATGGCCTACGC |
|  | **#5** | CACCGAGACCGAGCATCGGGGTAT | AAACATACCCCGATGCTCGGTCTC |
| **13. CBX8** | **#1** | CACCGCCGGTCCCTATCCCGGTCT | AAACAGACCGGGATAGGGACCGGC |
|  | **#2** | CACCGCCTCCTGAAGCGGCGCATA | AAACTATGCGCCGCTTCAGGAGGC |
|  | **#3** | CACCGGTGGGGGAGCGGGTGTTCG | AAACCGAACACCCGCTCCCCCACC |
|  | **#4** | CACCGTTTCCGTATGCGCCGCTTC | AAACGAAGCGGCGCATACGGAAAC |
|  | **#5** | CACCGCATGGAATACCTCGTGAAA | AAACTTTCACGAGGTATTCCATGC |
| **14. PHC1** | **#1** | CACCGTTGCAGGTAAACCGAACCC | AAACGGGTTCGGTTTACCTGCAAC |
|  | **#2** | CACCGCTGCTGCTGCTGCGCCAAC | AAACGTTGGCGCAGCAGCAGCAGC |
|  | **#3** | CACCGTTGTAGGCACTCGACAGCC | AAACGGCTGTCGAGTGCCTACAAC |
|  | **#4** | CACCGCTCAGTCAGCCCTGTCGGA | AAACTCCGACAGGGCTGACTGAGC |
|  | **#5** | CACCGCTGGGATCGGCTGATGAGC | AAACGCTCATCAGCCGATCCCAGC |
| **15. PHC2** | **#1** | CACCGGGCTGCACAGTAGCGACGG | AAACCCGTCGCTACTGTGCAGCCC |
|  | **#2** | CACCGGCTGGCTACCGCCCGGCGT | AAACACGCCGGGCGGTAGCCAGCC |
|  | **#3** | CACCGCCCTTCGATAACATGCGTC | AAACGACGCATGTTATCGAAGGGC |
|  | **#4** | CACCGTGGGATGCACCAAACGGGT | AAACACCCGTTTGGTGCATCCCAC |
|  | **#5** | CACCGATAACATGCGTCAGGATTT | AAACAAATCCTGACGCATGTTATC |
| **16. PHC3** | **#1** | CACCGTGGCTTGCCGTTAGGGTAG | AAACCTACCCTAACGGCAAGCCAC |
|  | **#2** | CACCGACGATGACGACGAGACAAC | AAACGTTGTCTCGTCGTCATCGTC |
|  | **#3** | CACCGTCAGATGAATGTGTCCGGA | AAACTCCGGACACATTCATCTGAC |
|  | **#4** | CACCGATCGTGGCCGTCGTCCAAG | AAACCTTGGACGACGGCCACGATC |
|  | **#5** | CACCGTGAGCTTGGCTTGCCGTTA | AAACTAACGGCAAGCCAAGCTCAC |
| **17. BCOR** | **#1** | CACCGGCTGTGAACGTTCCCATAC | AAACGTATGGGAACGTTCACAGCC |
|  | **#2** | CACCGACTGGAGAATACAGCGGCT | AAACAGCCGCTGTATTCTCCAGTC |
|  | **#3** | CACCGGTTCATCATGCCCGCGCAT | AAACATGCGCGGGCATGATGAACC |
|  | **#4** | CACCGGAGGGTTGGGTCCTCGTAA | AAACTTACGAGGACCCAACCCTCC |
|  | **#5** | CACCGCCAGTCTTCACCGTAAGCA | AAACTGCTTACGGTGAAGACTGGC |
| **18. SKP1** | **#1** | CACCGATTTGGGAATGGATGATGA | AAACTCATCATCCATTCCCAAATC |
|  | **#2** | CACCGCTGCATTCACATTTGGTAG | AAACCTACCAAATGTGAATGCAGC |
|  | **#3** | CACCGAGGAGGAGGGTCATCCTTG | AAACCAAGGATGACCCTCCTCCTC |
|  | **#4** | CACCGTTGTTCTCATCATCTTCAGG | AAACCCTGAAGATGATGAGAACAAC |
|  | **#5** | CACCGAACAGATGATATCCCTGTT | AAACAACAGGGATATCATCTGTTC |
| **19. L3MBTL2** | **#1** | CACCGCGATCCAGTACACCCGGCT | AAACAGCCGGGTGTACTGGATCGC |
|  | **#2** | CACCGGGCTACCTCATGAAACGGC | AAACGCCGTTTCATGAGGTAGCCC |
|  | **#3** | CACCGTGGGTTGGTCACGACGTGT | AAACACACGTCGTGACCAACCCAC |
|  | **#4** | CACCGGGTCTTGGGCTTCGACTGG | AAACCCAGTCGAAGCCCAAGACCC |
|  | **#5** | CACCGTGTGGTATCGTGGGTACAA | AAACTTGTACCCACGATACCACAC |
| **20.CBX3** | **#1** | CACCGTAGATCGACGTGTAGTGAA | AAACTTCACTACACGTCGATCTAC |
|  | **#2** | CACCGTAGATCGACGTGTAGTGAAT | AAACATTCACTACACGTCGATCTAC |
|  | **#3** | CACCGGAGCCTGAAGAATTTGTCG | AAACCGACAAATTCTTCAGGCTCC |
|  | **#4** | CACCGGAATATTTCCTGAAGTGGA | AAACTCCACTTCAGGAAATATTCC |
|  | **#5** | CACCGGACCTCTGGCAAATCCTCT | AAACAGAGGATTTGCCAGAGGTCC |
| **21. E2F6** | **#1** | CACCGCCAGCGATACATCAAAACG | AAACCGTTTTGATGTATCGCTGGC |
|  | **#2** | CACCGCTTGTCAGATCTGCTCCCG | AAACCGGGAGCAGATCTGACAAGC |
|  | **#3** | CACCGACATCACCAATGTCTTAGA | AAACTCTAAGACATTGGTGATGTC |
|  | **#4** | CACCGATCAGCAATGGAAGATGCTT | AAACAAGCATCTTCCATTGCTGATC |
|  | **#5** | CACCGGCTCCAGCAGAAACCAGAT | AAACATCTGGTTTCTGCTGGAGCC |
| **22. KDM2B** | **#1** | CACCGCTCGTTCTCGTCGTATCGC | AAACGCGATACGACGAGAACGAGC |
|  | **#2** | CACCGAGGAGATCGTCAGCGTCCG | AAACCGGACGCTGACGATCTCCTC |
|  | **#3** | CACCGGGGGACTTCGTGCACGCCA | AAACTGGCGTGCACGAAGTCCCCC |
|  | **#4** | CACCGTTGGCCGCCAACCGGACAA | AAACTTGTCCGGTTGGCGGCCAAC |
|  | **#5** | CACCGGCTCCTGCCGTTGTCCGGT | AAACACCGGACAACGGCAGGAGCC |
| **23. RYBP** | **#1** | CACCGCGAAACCTGCCGCAGACGA | AAACTCGTCTGCGGCAGGTTTCGC |
|  | **#2** | CACCGGAAACCTGCCGCAGACGAA | AAACTTCGTCTGCGGCAGGTTTCC |
|  | **#3** | CACCGCCAGCTGAGAATTGATCCG | AAACCGGATCAATTCTCAGCTGGC |
|  | **#4** | CACCGTGAGGTGTGATTTGTTTCGC | AAACGCGAAACAAATCACACCTCAC |
|  | **#5** | CACCGTCCCAAAACCCTTCGTCTG | AAACCAGACGAAGGGTTTTGGGAC |

**Supplementary Table 3**

A list of primers to detect the expression of PRC1 genes for the CRISPR/Cas9 screening

| **Target gene** | **5’-forward primer** | **5’-reverse primer** |
| --- | --- | --- |
| **1. PCGF1** | ATTGTGAAGTACCTCCAAACTAGCA | TTGAGCAGTGGCTGTGTCTC |
| **2. PCGF2** | GAGTGCCTGCATTCCTTCTG | CACACATGGGGCAGTATTTG |
| **3. PCGF3** | ACATCAACGCCCACATCAC | GTGTGCAGACACTCGGTCAC |
| **4. PCGF4** | TCAAGCAGAAATGCATCGAA | CACAAAGCACACACATCAGGT |
| **5. PCGF5** | CCAAAGGAAACACTTGGTGAA | TGTTGGCTTGATCAGATACCC |
| **6. PCGF6** | TGGTGTCTCTCCCGACCAT | TGCAGCTCCCTCGGTTTT |
| **7. RING1A** | GAATACGAGGCCCATCAAGA | AGCTCAATGCCTGCTGGT |
| **8. RING1B** | TGAGTTACAACGAACACCTCAGG | TAGACTTCGAGGTGAAACCACA |
| **9. CBX2** | CGAGTGCATCCTGAGCAA | GCTGTTATGTTTGGAGGACCA |
| **10. CBX4** | GGTCGCCCAAATATAACACG | CTGTTCTGGAAGGCGATCA |
| **11. CBX6** | CCCAAAACTTTCCTCCTGAAG | CGGCTTGACAGAGAAATGC |
| **12. CBX7** | CGTGCGGAAGGGTAAAGTC | CTCCCACGTGCTGTACTTTG |
| **13. CBX8** | ATACGGAAAGGACGCATGG | CGGTTCCCATGTGCTGTACT |
| **14. PHC1** | GCAGATGCAGATCAGGTTCA | CCTTGCATCTGAGGTCCTTG |
| **15. PHC2** | GAAACTCTGCCTCCAGCATC | GGATTTGGGGTTTCACAATG |
| **16. PHC3** | CTCCGAGCTCAAATGCTGAT | ACGATGACGACGAGACAACA |
| **17. BCOR** | CCCAGAACCGAGTTTCAAAG | ACCTGAATGCCTCATTTGGA |
| **18. SKP1** | AAGACCATGTTGGAAGATTTGG | TTGCTGCATTCACATTTGGT |
| **19. L3MBTL2** | TGTGCCATCAACAGCAAGAT | TCATGAGGTAGCCCTTCCAG |
| **20. CBX3** | GTGTCTATAATTGCAGTGGTTTATTTG | ACACACTAGCATTTGTCTAGTTTCCTC |
| **21. E2F6** | CAATGGAAGATGCTTTGGATGAG | GCTGGAGCTTTAACTGCAATG |
| **22. KDM2B** | TGGAGGGCAAAGATTTCAAC | TCTCGAAATATCAGGGGAACC |
| **23. RYBP** | CGAAGGGTTTTGGGATTGTA | GCCACCAGCTGAGAATTGAT |

**Supplementary Method S1**

*Construction of the modified plasmid, pLX-sgRNA ver.2*

We have modified the pLX-sgRNA plasmid (Addgene plasmid #50662) so that we can easily clone each of the annealed oligonucleotides for sgRNAs into the restriction enzyme sites. At first, we disrupted the BsmBI site in the original pLX-sgRNA plasmid. The DNA fragment was amplified by PCR with the following primer set, 5’-TAAGACCACCGCACAGCAAG-3’ and 5’-ACTAGTAAGACGTGCGGCTTCCGTTTG-3’ using pLX-sgRNA as a template. The amplified fragment was digested with NotI and SpeI (Takara, Ohtsu, Japan), and then cloned into the corresponding region between NotI and SpeI site of the original pLX-sgRNA plasmid. Next we cloned the DNA fragment containing human U6 promoter, scaffold sequences of sgRNA and BsmBI sites from the lentiGuide-Puro plasmid (Addgene plasmid #52963) using the following primer set, 5’-CTCGAGTTTCCCATGATTCCTTCATATTTG-3’ and 5’-GCTAGCAAAAAAGCACCGACTCG-GTGC-3’. The amplified fragment was digested with XhoI and NheI, and then cloned into the corresponding region between XhoI and NheI site of the BsmBI-disrupted pLX-sgRNA plasmid. We named the modified plasmid pLX-sgRNA ver.2 (Fig. S1A).


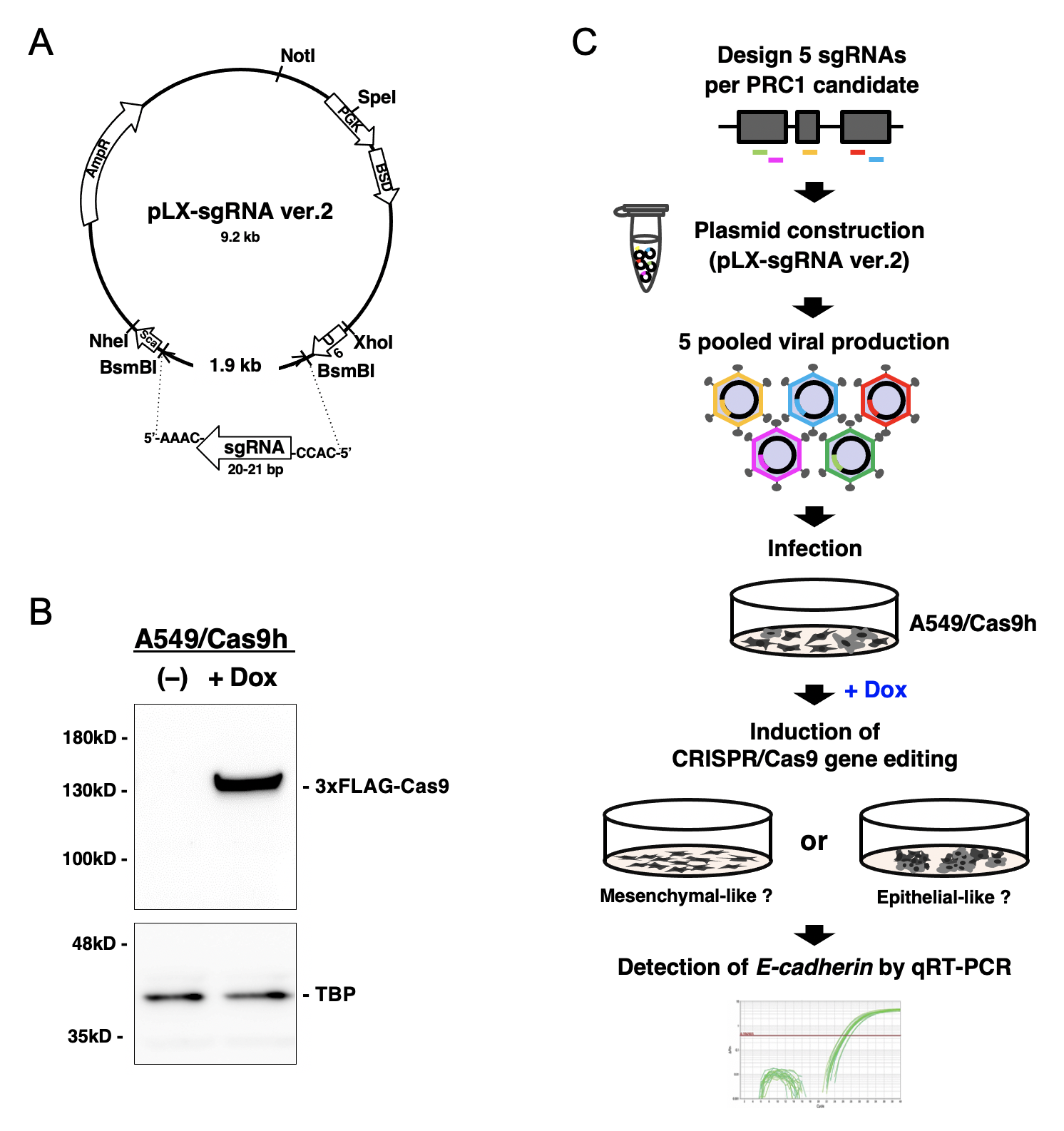


**Supplementary Fig. S1.** The workflow of CRISPR/Cas9 screening to identify candidate PRC1 members related to EMT process.

(A) The modified plasmid, pLX-sgRNA ver.2, for sgRNA expression in this study. The annealed oligonucleotides for each sgRNA were cloned into the vector digested with BsmBI. The construction of pLX-sgRNA ver.2 was described in Supplementary Method 1. The abbreviations: PGK, human phosphoglycerate kinase promoter; BSD, blasticidin S deaminase; U6, RNA polymerase III promoter for human U6 snRNA; Sca, guide RNA scaffold; AmpR, ampicillin resistant gene. (B) Immunoblot analysis to confirm the induced expression of Cas9 proteins. A549/Cas9h cells were treated with 10 μg/ml doxycycline (Dox) for 48 hours, and 3xFLAG-Cas9 proteins were detected by anti-FLAG antibody. (C) Experimental workflow of the CRISPR/Cas9 screening for candidate PRC1-related genes.


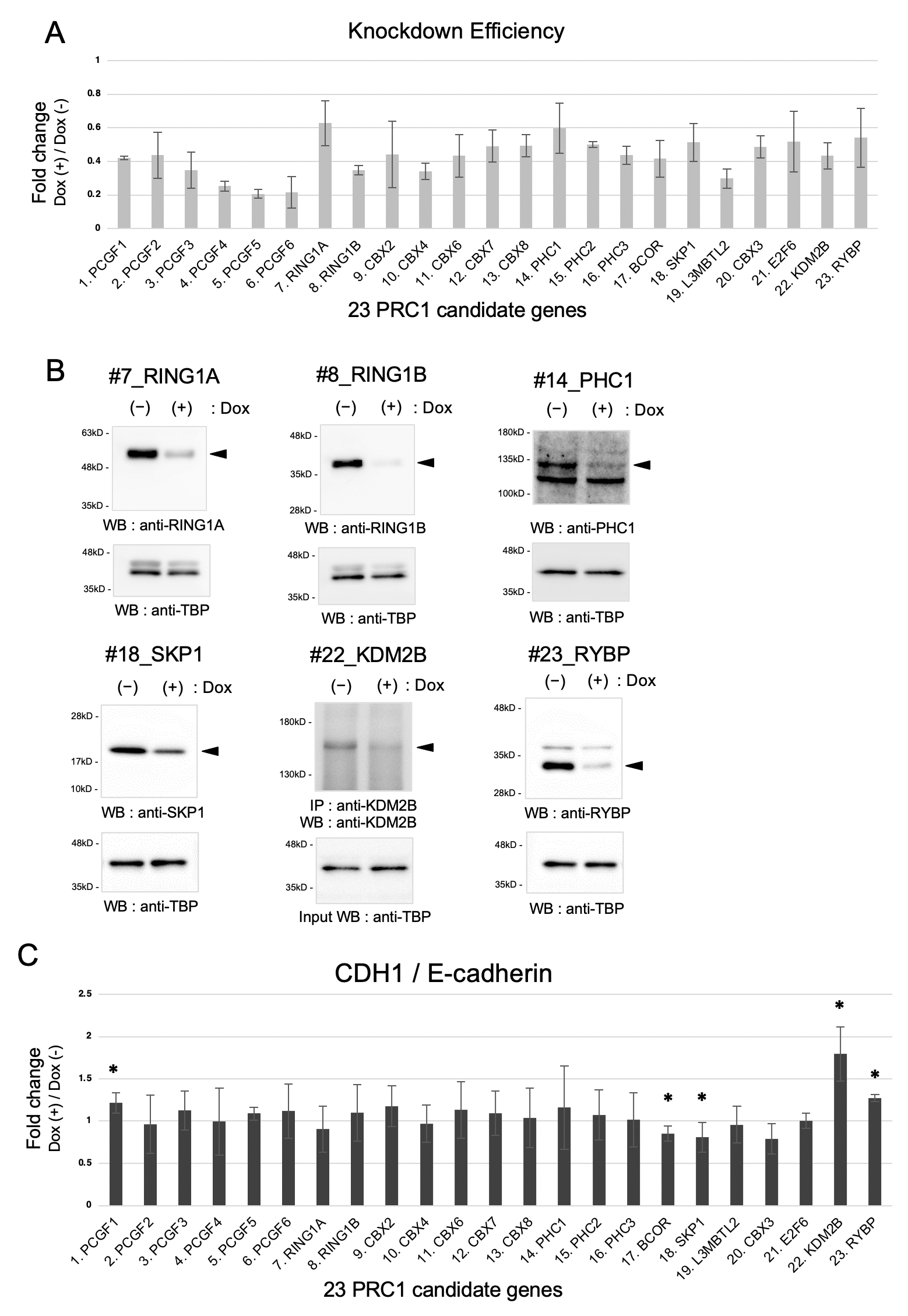


**Supplementary Fig. S2.** The results of CRISPR/Cas9 screening to identify candidate PRC1 members related to EMT process.

1. The expression of each PRC1-related gene in A549/Cas9h cells introduced with its five sgRNAs.

The expression of each gene was quantified by QRT-PCR in the control and doxycycline-treated cells and the fold changes were presented with the standard deviations (n=3). The downregulation of each gene was statistically significant (*P* < 0.05). (B) Immunoblotting of RING1A, RING1B, PHC1, SKP1, KDM2B and RYBP proteins using the corresponding antibodies in the sgRNA-introduced A549/Cas9h cells with or without doxycycline. For endogenous KDM2B detection, we performed the immunoprecipitation followed by immunoblotting. The corresponding bands of the proteins were indicated by the black arrow heads. The blots of TBP were shown as loading control. (C) The expression of *CDH1/E-cadherin* (an epithelial marker gene) in A549/Cas9h cells introduced with the sgRNAs for each gene. The expression of *CDH1* was measured by QRT-PCR and the fold changes were presented with the standard deviations (n=3) (*, *P* < 0.05).

**
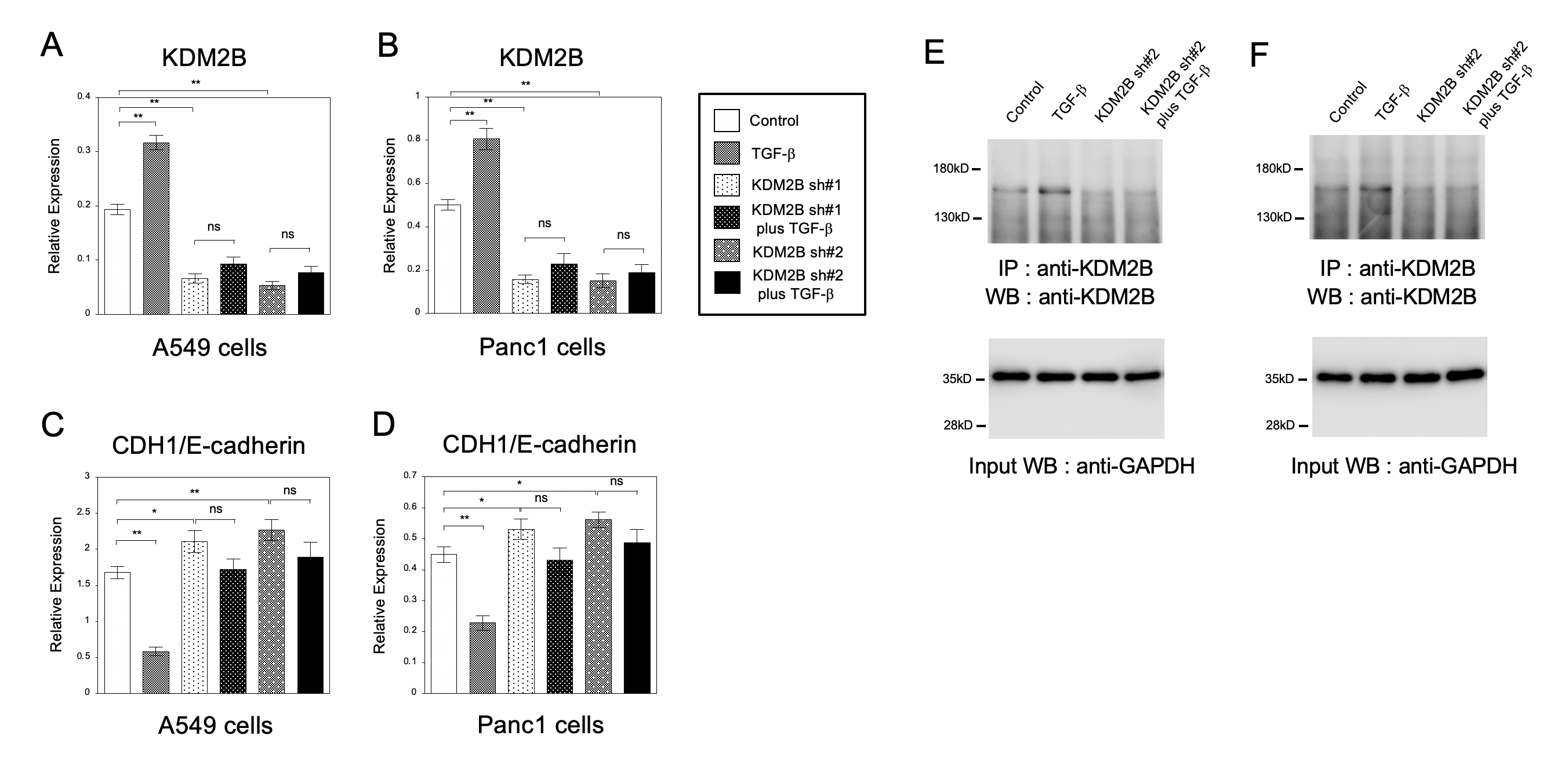
**

**Supplementary Fig. S3.** The expression of *KDM2B* and *CDH1* in A549 and Panc1 cells with *KDM2B* knockdown by shRNA.

QRT-PCR was performed to detect the expression of *KDM2B* (A)(B) or *CDH1/E-cadherin* (C)(D) in A549 cells (A)(C) and Panc1 (B)(D) cells infected with lentivirus expressing control shRNA, *KDM2B* shRNA#1 or shRNA #2 without or with TGF-β treatment for 24 hours (n=3) (**, *P* < 0.01; *, *P* < 0.05; ns, not significant). (E)(F) Immunoprecipitation followed by immunoblotting of endogenous KDM2B proteins in A549 cells (E) and Panc1 (F) cells with *KDM2B* knockdown (*KDM2B* shRNA#2) without or with TGF-β treatment. For the control, GAPDH proteins in the input cell lysates were detected by immunoblotting. The pictures for GAPDH proteins are common with those in the bottom panels of Fig. 1C and 1D.


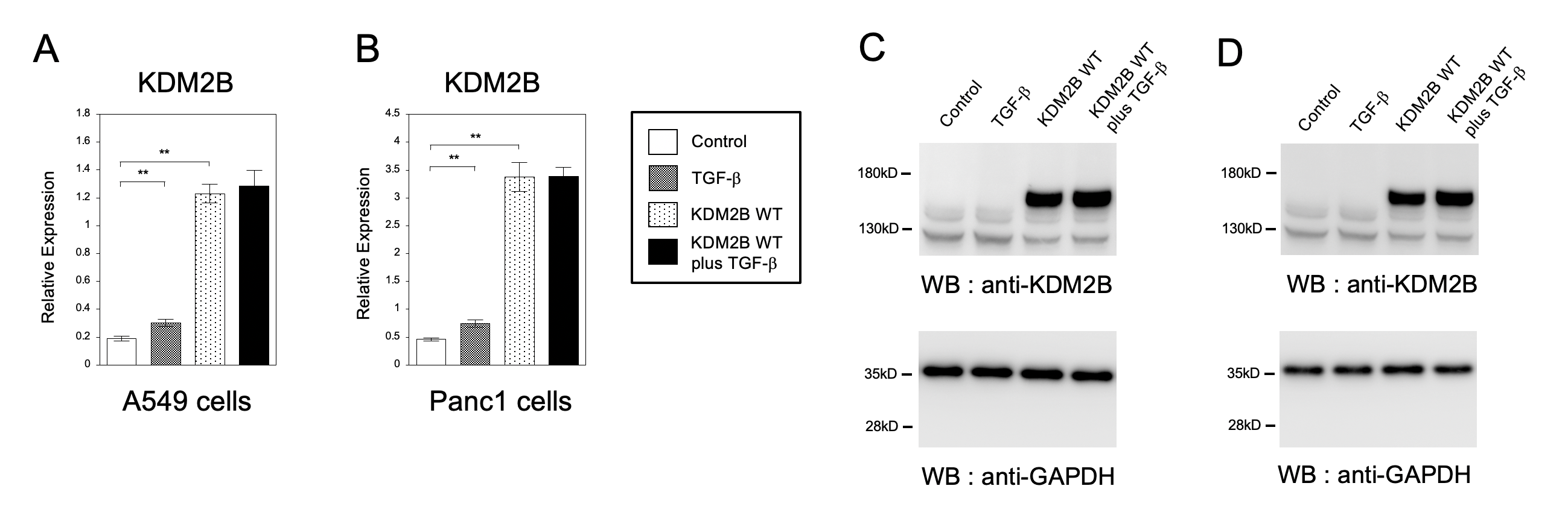


**Supplementary Fig. S4.** The expression of *KDM2B* in A549 and Panc1 cells with *KDM2B* overexpression.

QRT-PCR was performed to detect the expression of *KDM2B* in A549 cells (A) and Panc1 (B) cells infected with the control retrovirus or the retrovirus expressing wildtype *KDM2B* (WT) with or without TGF-β treatment for 24 hours (n=3) (**, *P* < 0.01). (C)(D) Immunoblotting of KDM2B and GAPDH proteins in A549 cells (C) and Panc1 (D) cells with *KDM2B* overexpression without or with TGF-β treatment. In the simple immunoblotting, endogenous KDM2B proteins were not detected with the KDM2B antibody. The pictures for GAPDH are common with those in the bottom panels of Fig. 2C and 2D.


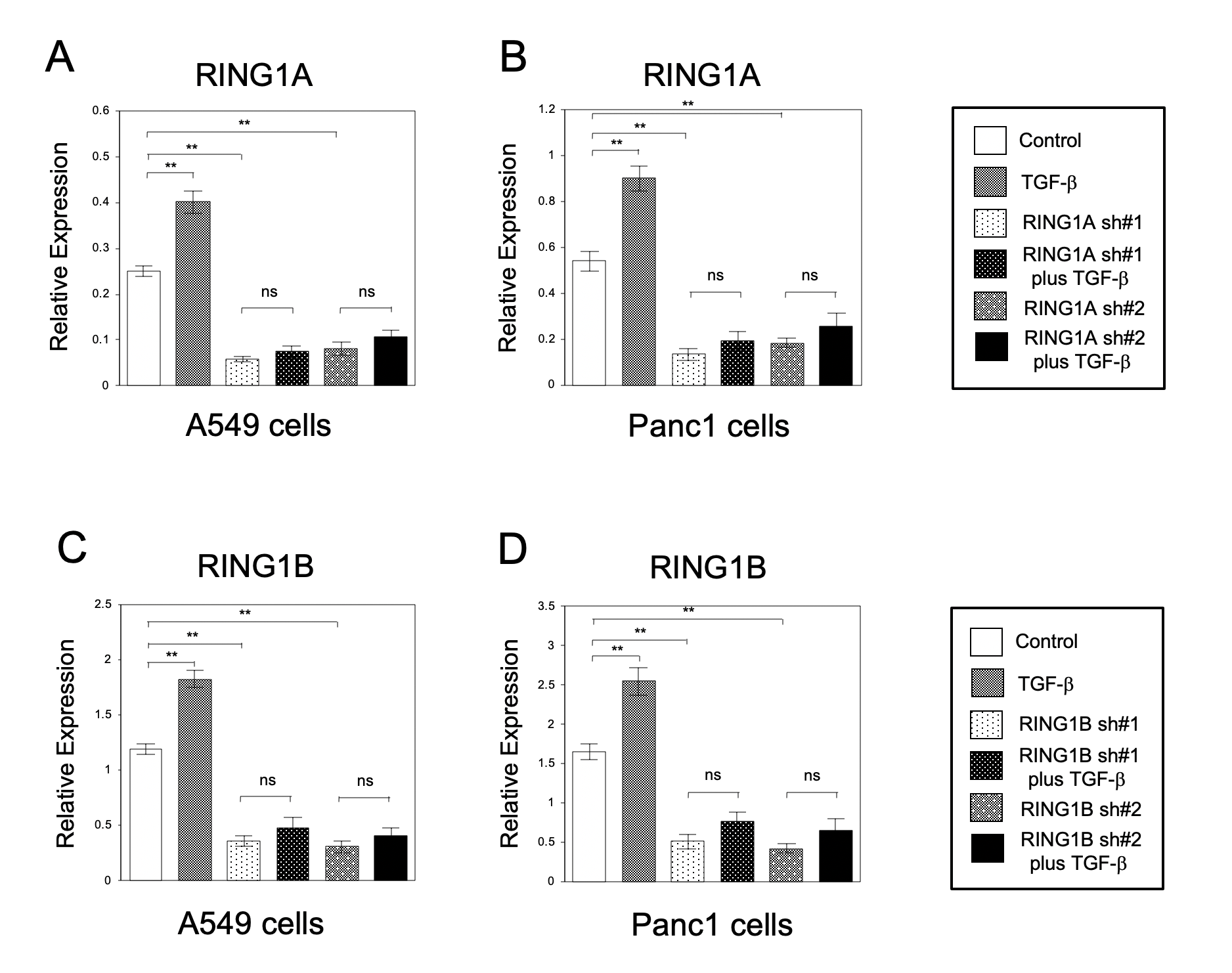


**Supplementary Fig. S5.** The expression of *RING1A* and *RING1B* in A549 and Panc1 cells with knockdown of each gene by shRNA.

QRT-PCR was performed to detect the expression of *RING1A* (A)(B) or *RING1B* (C)(D) in A549 cells (A)(C) and Panc1 (B)(D) cells infected with lentivirus expressing control shRNA, *RING1A* shRNA#1, *RING1A* shRNA #2, *RING1B* shRNA#1 or *RING1B* shRNA#2 without or with TGF-β treatment (n=3) (**, *P* < 0.01; ns, not significant).


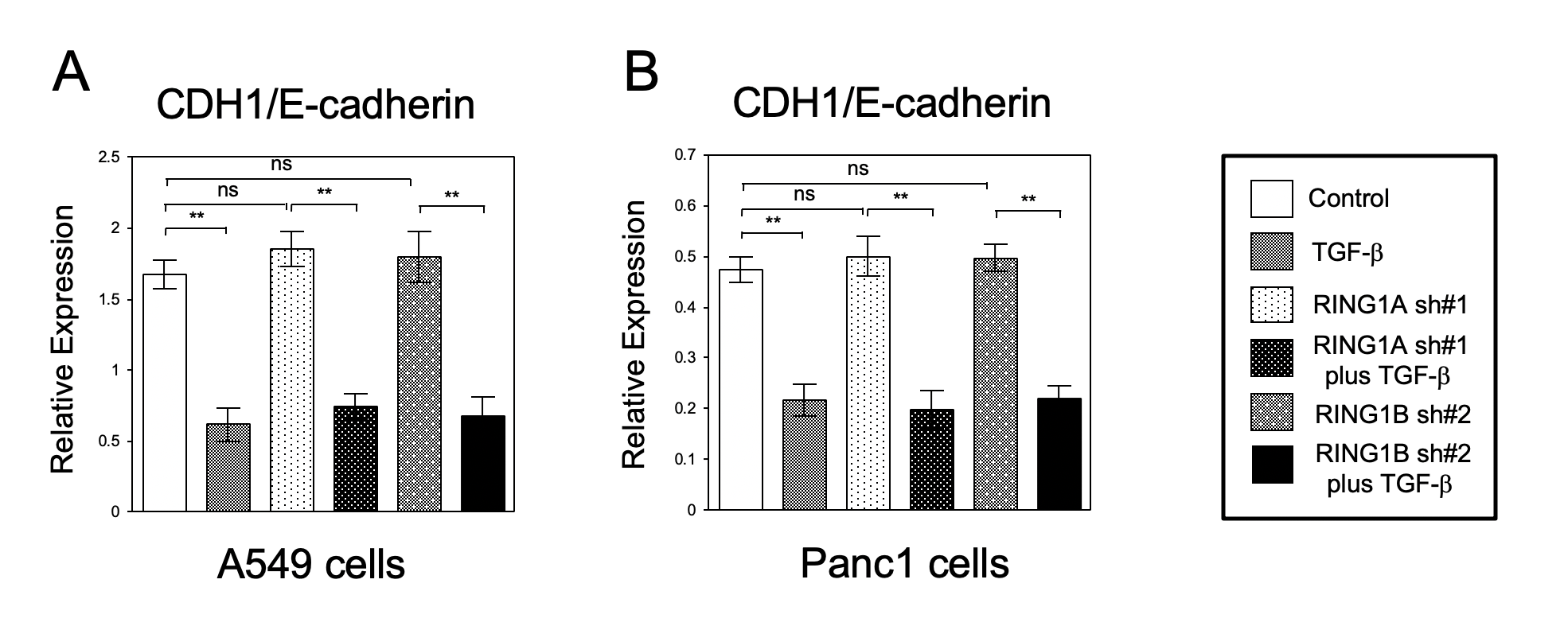


**Supplementary Fig. S6.** The expression of *CDH1/E-cadherin* in A549 and Panc1 cells with either *RING1A* or *RING1B* knockdown.

QRT-PCR was performed to detect the expression of *CDH1/E-cadherin* (A)(B) in A549 cells (A) and Panc1 (B) cells infected with lentivirus expressing control shRNA, *RING1A* shRNA#1 or *RING1B* shRNA #2 without or with TGF-β treatment (n=3) (**, *P* < 0.01; ns, not significant).


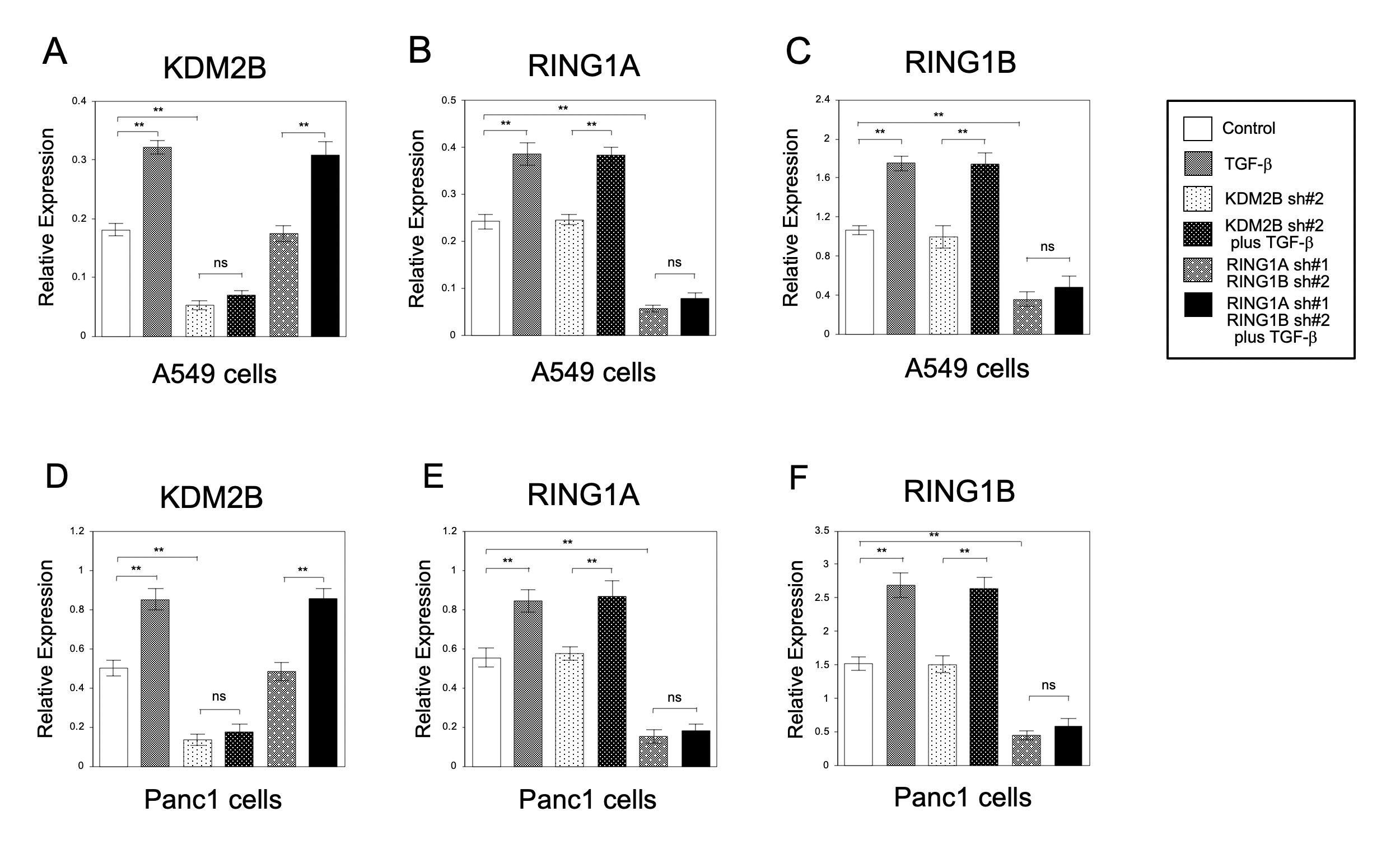


**Supplementary Fig. S7.** The expression of *KDM2B*, *RING1A* and *RING1B* in A549 and Panc1 cells with *KDM2B* knockdown or *RING1A/RING1B* combined knockdown.

QRT-PCR was performed to detect the expression of *KDM2B*(A)(D), *RING1A* (B)(E) or *RING1B* (C)(F) in A549 cells (A)(B)(C) and Panc1 (D)(E)(F) cells infected with lentivirus expressing control shRNA, *KDM2B* shRNA#2 or *RING1A* shRNA #1/*RING1B* shRNA#2 without or with TGF-β treatment (n=3) (**, *P* < 0.01; ns, not significant).


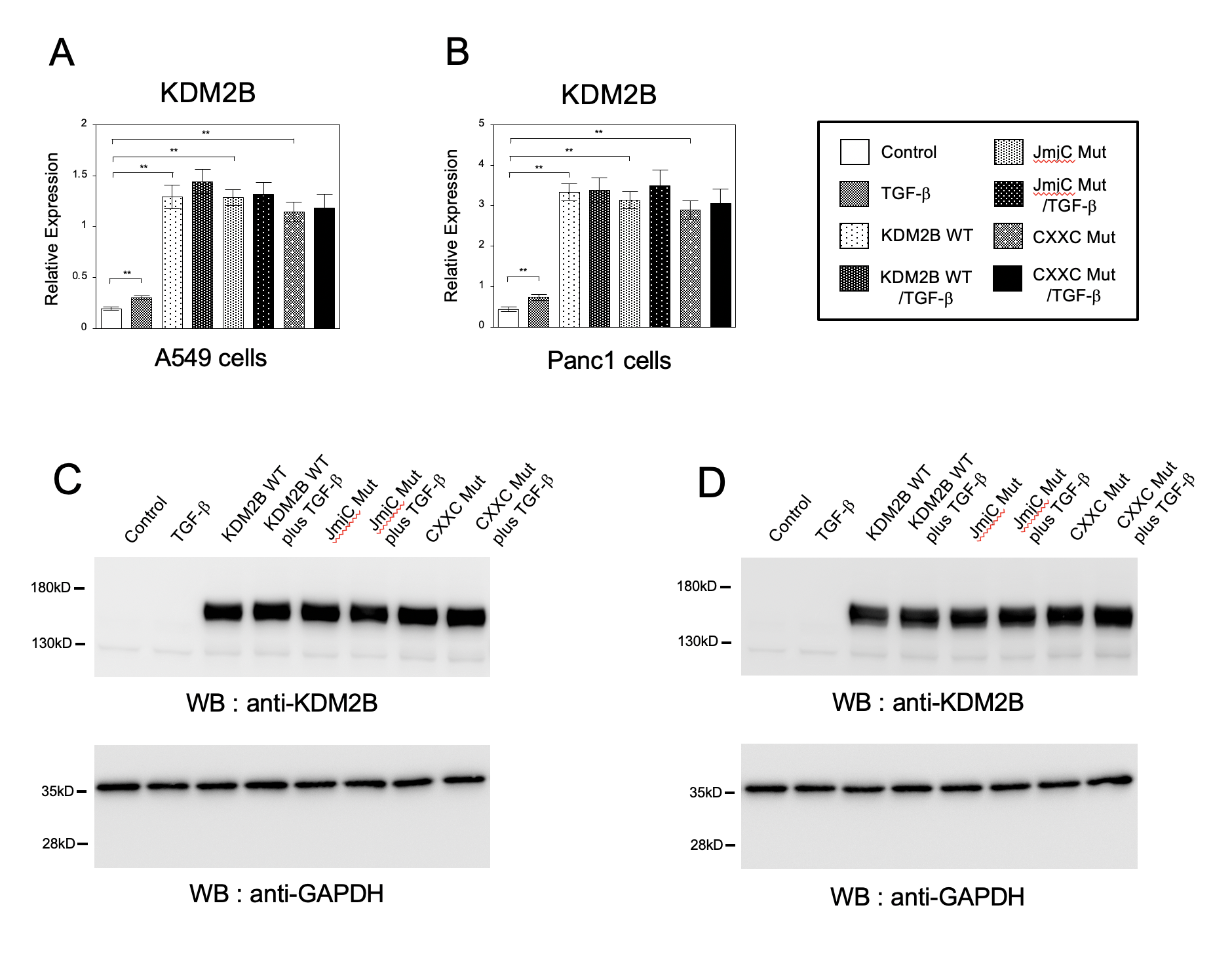


**Supplementary Fig. S8.** The expression of *KDM2B* in A549 and Panc1 cells with overexpression of wildtype and mutant *KDM2B*.

QRT-PCR was performed to detect the expression of *KDM2B* in A549 cells (A) and Panc1 (B) cells infected with the control retrovirus or the retrovirus expressing wildtype *KDM2B* (WT) , JmjC domain mutant (JmjC Mut) or CXXC motif mutant (CXXC Mut) with or without TGF-β treatment for 24 hours (n=3) (**, *P* < 0.01). (C)(D) Immunoblotting of KDM2B and GAPDH proteins in A549 cells (C) and Panc1 (D) cells with wildtype and mutant *KDM2B* overexpression without or with TGF-β treatment. The pictures for GAPDH are common with those in the bottom panels of Fig. 5C and 5D.


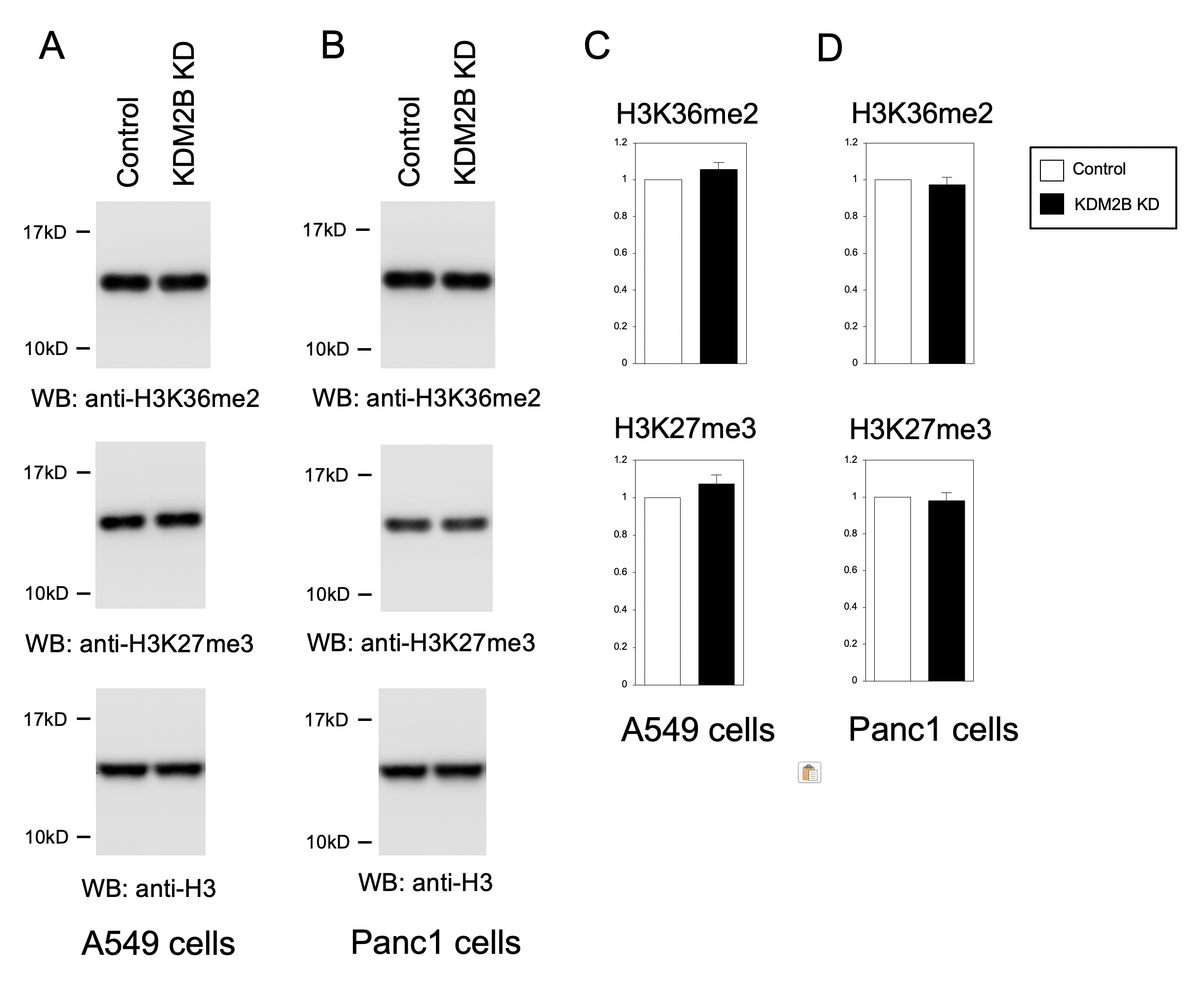


**Supplementary Fig. S9.** The effect of *KDM2B* knockdown on the overall levels of histone H3K36 and H3K27 methylation in A549 and Panc1 cells.

(A)(B) Immunoblotting was performed to detect the overall levels of histone H3K36 and H3K27 methylation in A549 (A) and Panc1 (B) cells infected with the control lentivirus or the lentivirus expressing *KDM2B* shRNA#2 (KD). The representative results were shown by using anti-H3K36me2 (upper panel), anti-H3K27me3 (middle panel) and anti-histone H3 antibodies (lower panel) for immunoblotting. (C)(D) The quantified data of overall levels of histone H3 methylation in A549 (C) and Panc1 (D) cells. The quantified data for methylated histones were normalized with control histone H3 expression. The averages from at least three independent experiments are shown with the standard deviations (n=3). There is no statistically significant difference.


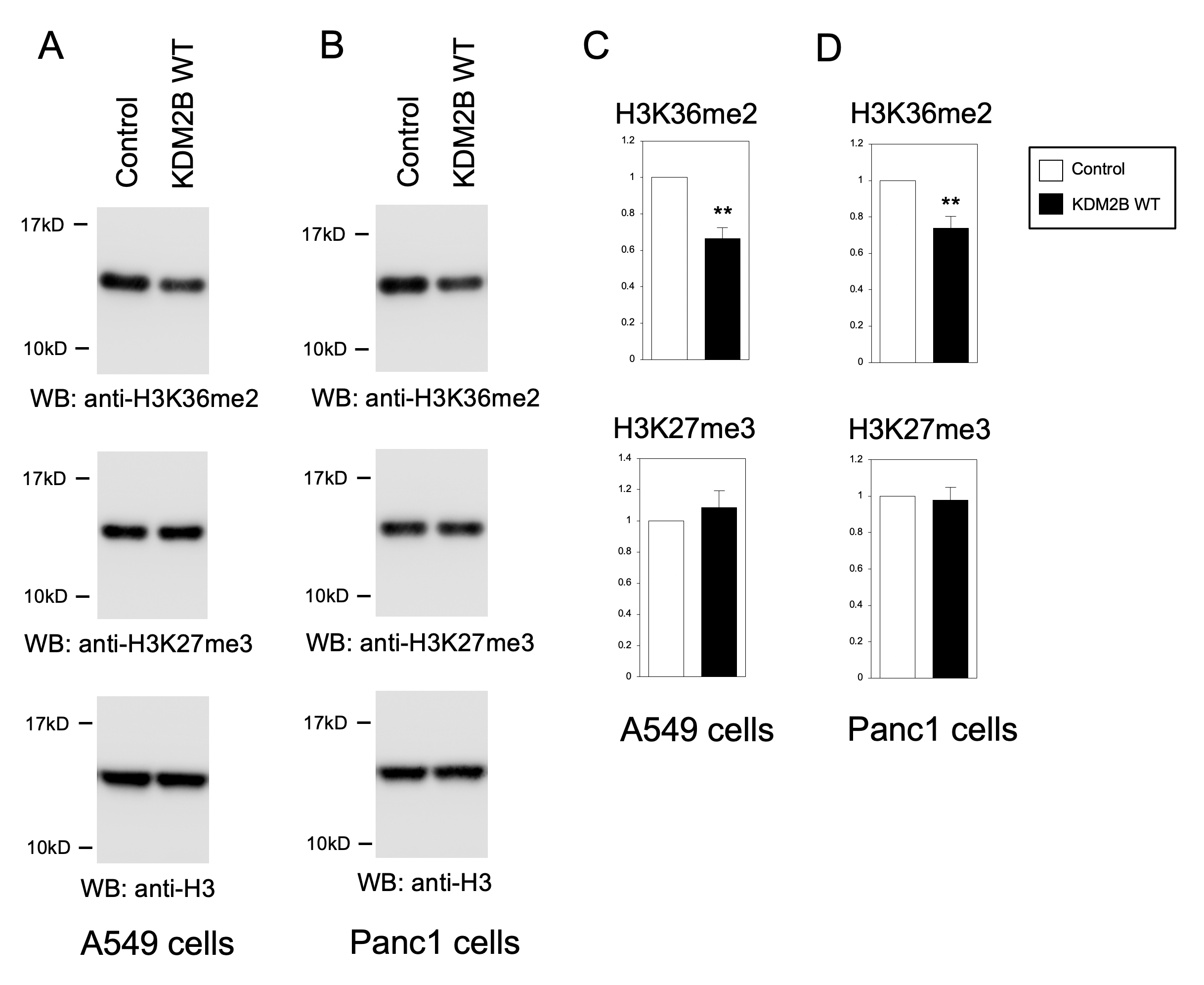


**Supplementary Fig. S10.** The effect of *KDM2B* overexpression on the overall levels of histone H3K36 and H3K27 methylation in A549 and Panc1 cells.

(A)(B) Immunoblotting was performed to detect the overall levels of histone H3K36 and H3K27 methylation in A549 (A) and Panc1 (B) cells infected with the control retrovirus or the retrovirus expressing wildtype *KDM2B* (WT). The representative results were shown by using anti-H3K36me2 (upper panel), anti-H3K27me3 (middle panel) and anti-histone H3 antibodies (lower panel) for immuno-blotting. (C)(D) The quantified data of overall levels of histone H3 methylation in A549 (C) and Panc1 (D) cells. The quantified data for methylated histones were normalized with control histone H3 expression. The averages from at least three independent experiments are shown with the standard deviations (n=3) (**, *P* < 0.01).


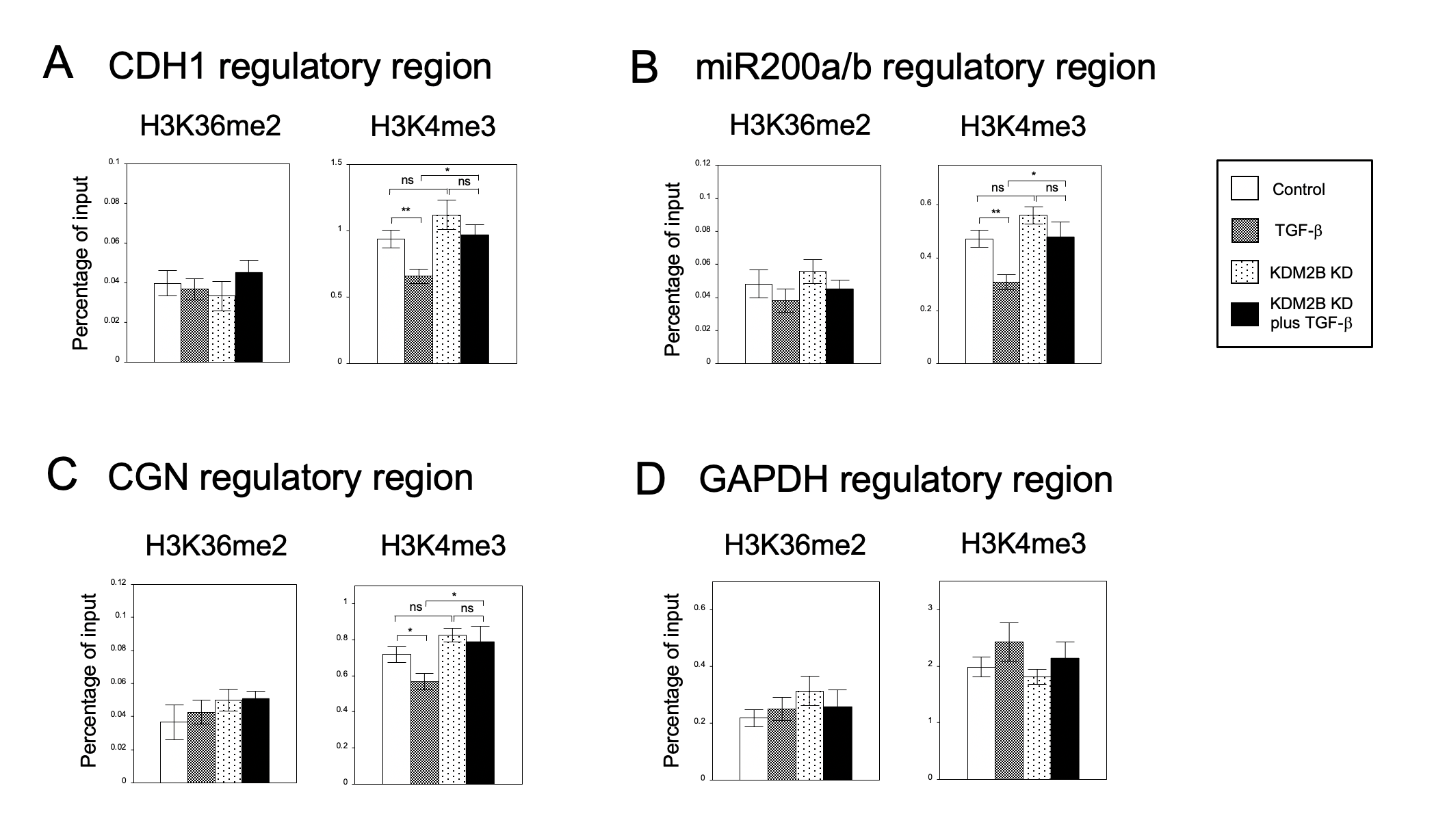


**Supplementary Fig. S11.** The effects of *KDM2B* knockdown in the regulation of histone H3K36 and H3K4 methylation on the regulatory regions of several epithelial marker genes in A549 cells.

A549 cells were infected with the control lentivirus or the lentivirus expressing *KDM2B* shRNA#2 (KD) with or without TGF-β treatment. ChIP analyses of H3K36me2 and H3K4me3 on the regulatory regions of *CDH1* (A), *miR200a/b* (B), *CGN* (C) and *GAPDH* genes (D) in A549 cells are shown. The occupancies of methylated histones on the regions were analyzed by quantitative PCR. Percentage enrichment over input chromatin DNA was presented (n=3) (**, *P* < 0.01; *, *P* < 0.05; ns, not significant).


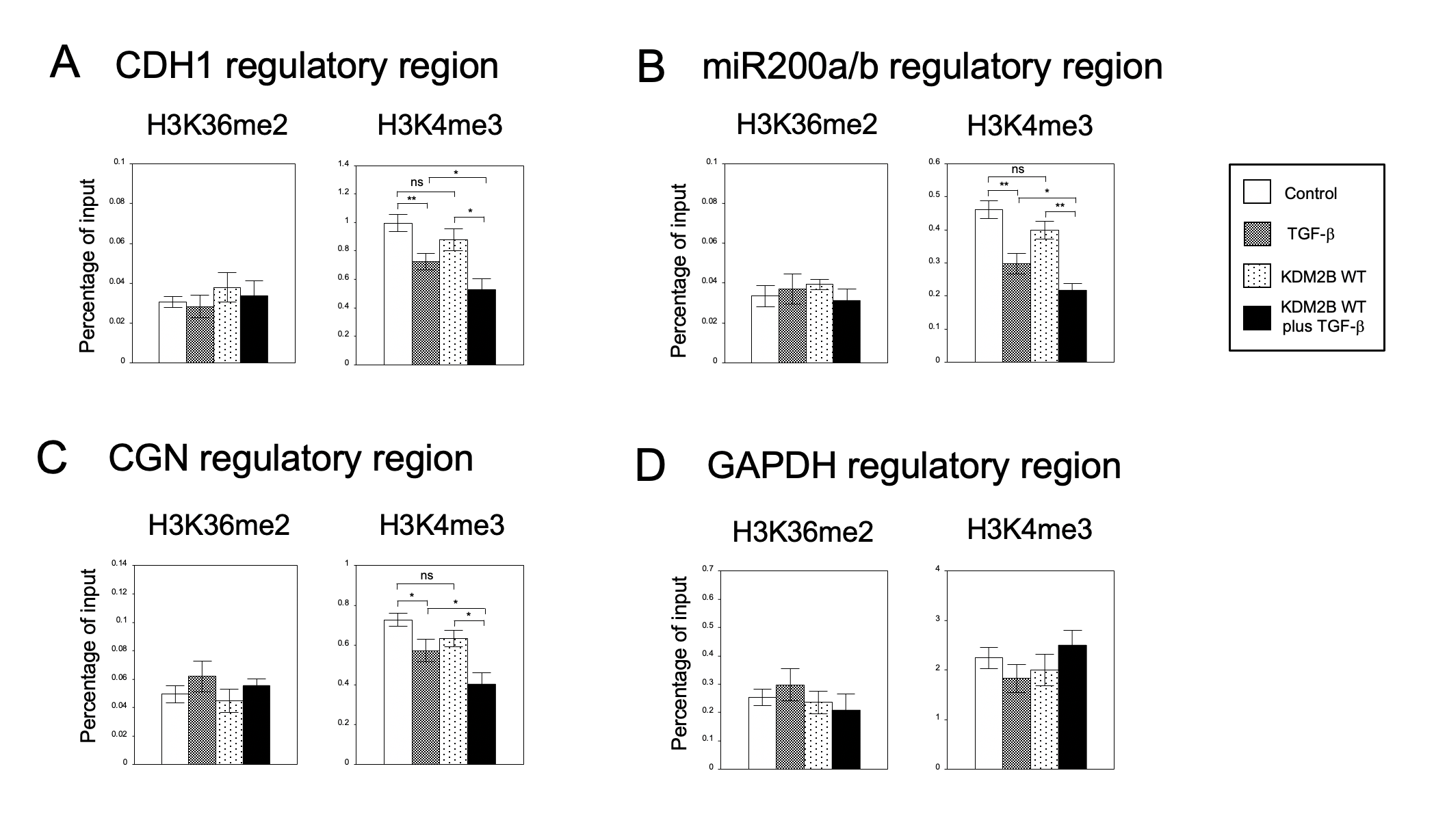


**Supplementary Fig. S12.** The effects of *KDM2B* overexpression in the regulation of histone H3K36 and H3K4 methylation on the regulatory regions of several epithelial marker genes in A549 cells.

A549 cells were infected with the control retrovirus or the retrovirus expressing wildtype *KDM2B* (WT) with or without TGF-β treatment. ChIP analyses of H3K36me2 and H3K4me3 on the regulatory regions of *CDH1* (A), *miR200a/b* (B), *CGN* (C) and *GAPDH* genes (D) in A549 cells are shown. The occupancies of methylated histones on the regions were analyzed by quantitative PCR. Percentage enrichment over input chromatin DNA was presented (n=3) (**, *P* < 0.01; *, *P* < 0.05; ns, not significant).


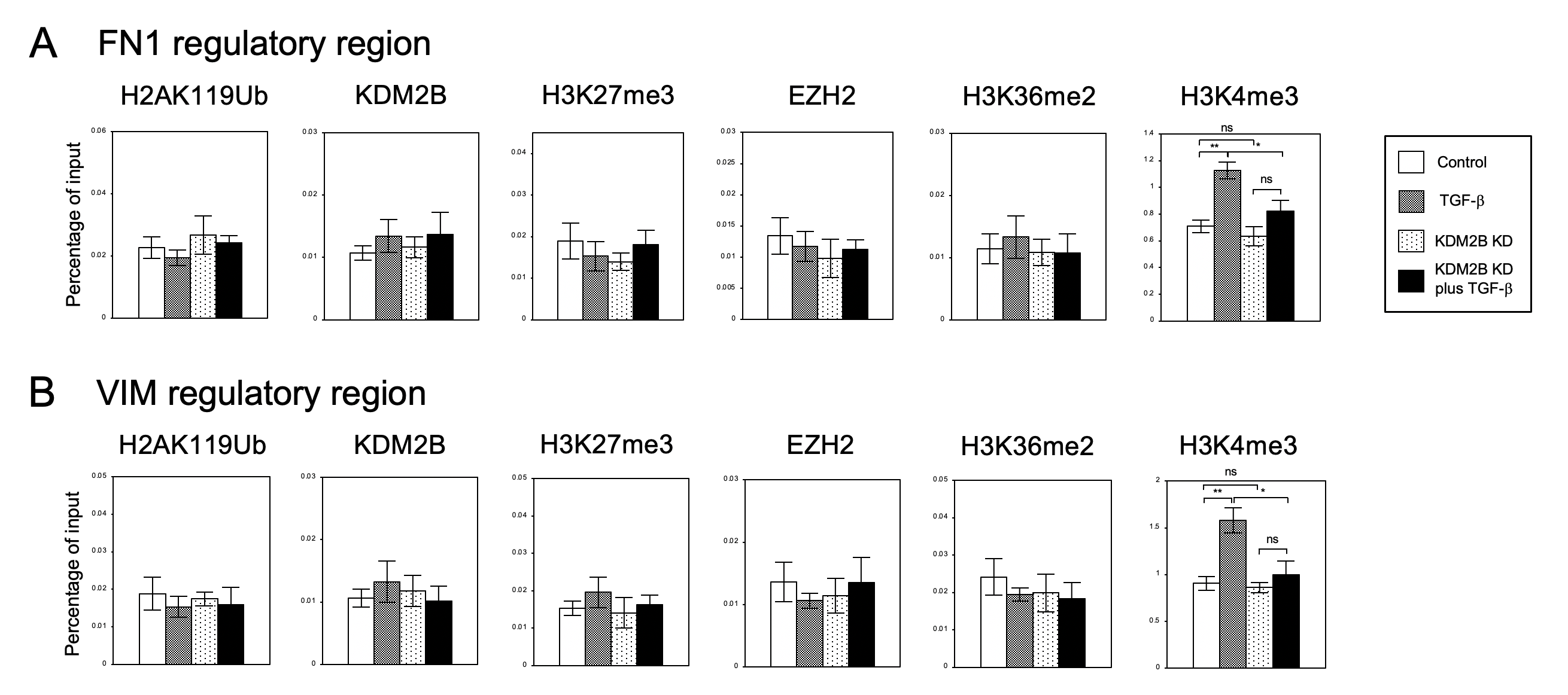


**Supplementary Fig. S13.** The effects of *KDM2B* knockdown in the regulation of histone H2A ubiquitination and H3 methylation on the regulatory regions of mesenchymal marker genes, *FN1* and *VIM* in A549 cells.

A549 cells were infected with the control lentivirus or the lentivirus expressing *KDM2B* shRNA#2 (KD) with or without TGF-β treatment. ChIP analyses of H2AK119Ub, KDM2B, H3K27me3, EZH2, H3K36me2 and H3K4me3 on the regulatory regions of *FN1* (A) and *VIM* genes (B) in A549 cells are shown. The occupancies of ubiquitinated histones, KDM2B, methylated histones or EZH2 proteins on the regions were analyzed by quantitative PCR. Percentage enrichment over input chromatin DNA was presented (n=3) (**, *P* < 0.01; *, *P* < 0.05; ns, not significant).


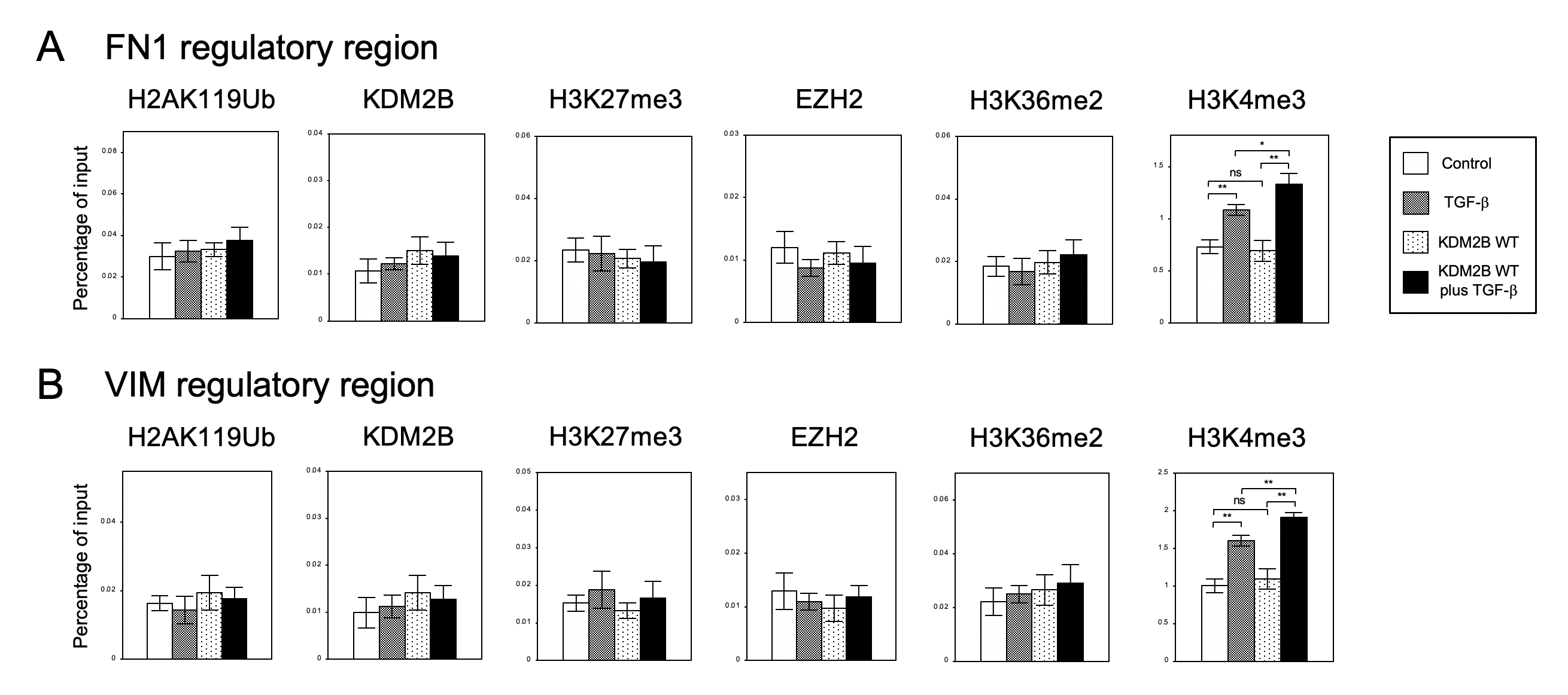


**Supplementary Fig. S14.** The effects of *KDM2B* overexpression in the regulation of histone H2A ubiquitination and H3 methylation on the regulatory regions of mesenchymal marker genes, *FN1* and *VIM* in A549 cells.

A549 cells were infected with the control retrovirus or the retrovirus expressing wildtype *KDM2B* (WT) with or without TGF-β treatment. ChIP analyses of H2AK119Ub, KDM2B, H3K27me3, EZH2, H3K36me2 and H3K4me3 on the regulatory regions of *FN1* (A) and *VIM* genes (B) in A549 cells are shown. The occupancies of ubiquitinated histones, KDM2B, methylated histones or EZH2 proteins on the regions were analyzed by quantitative PCR. Percentage enrichment over input chromatin DNA was presented (n=3) (**, *P* < 0.01; *, *P* < 0.05; ns, not significant).


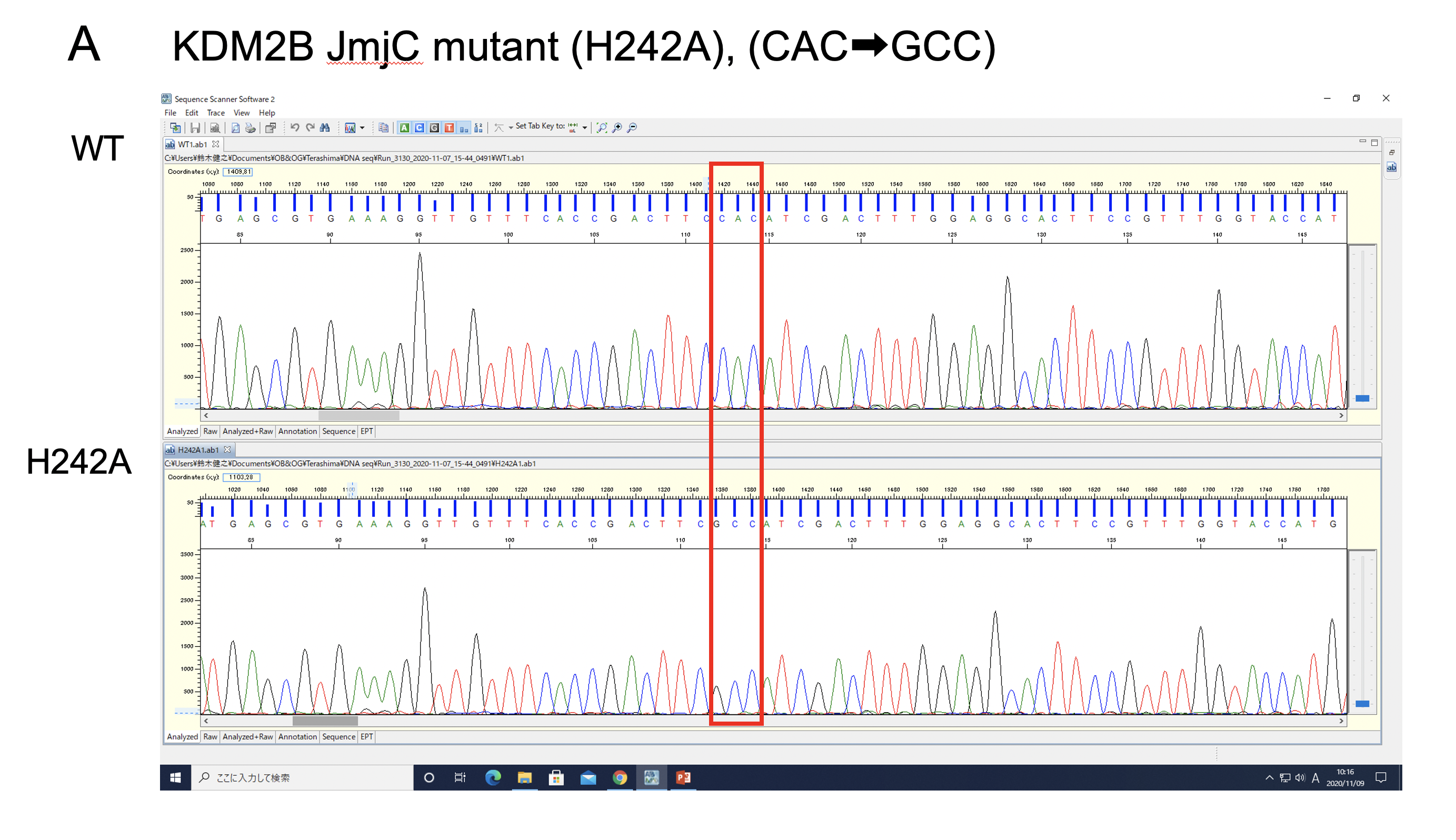


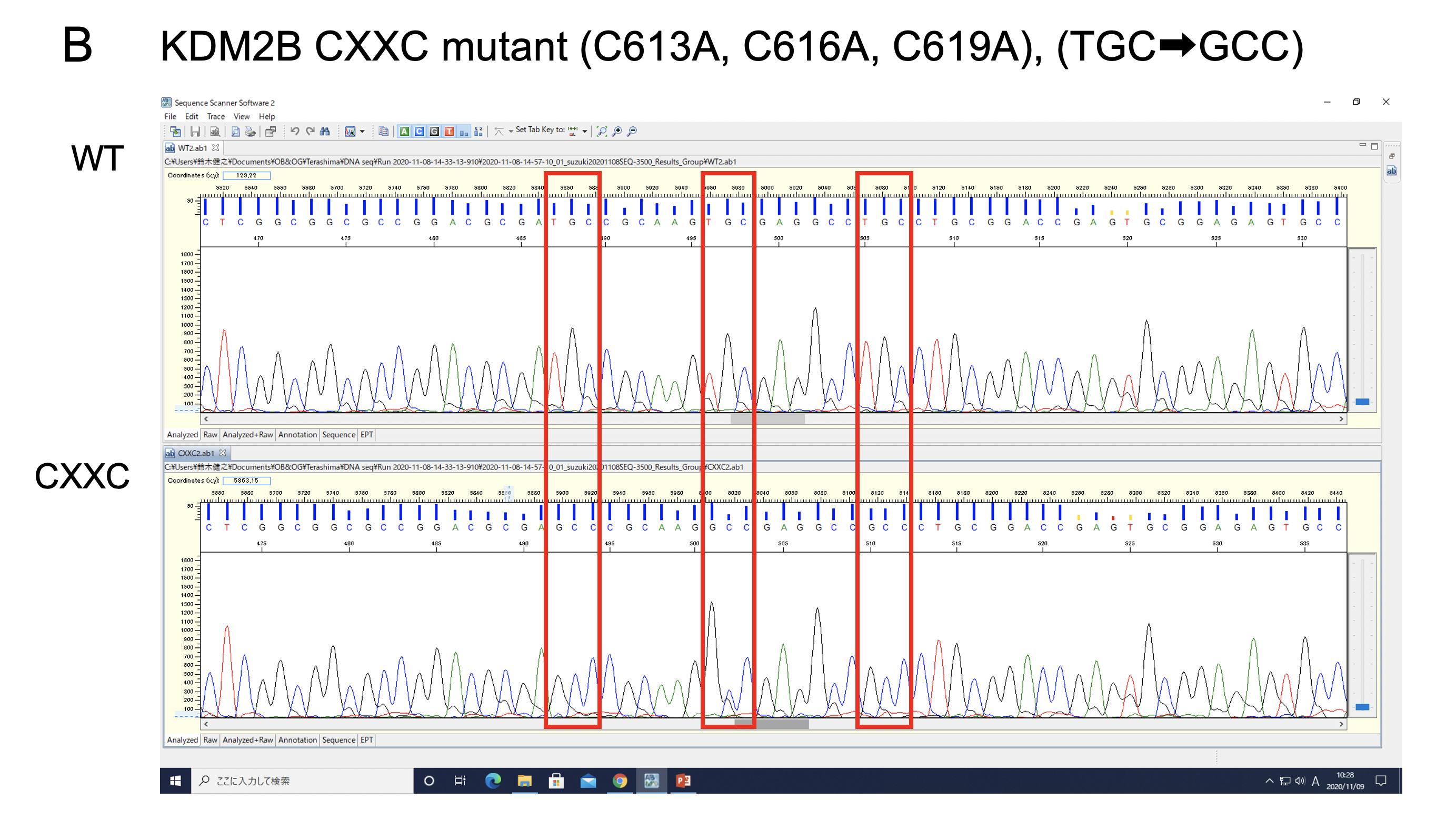


**Supplementary Fig. S15.** The nucleotide exchanges of KDM2B mutants confirmed by DNA sequencing.

DNA sequences of KDM2B JmjC mutant (A) and CXXC mutant (B) in the expression vectors were determined and compared with the sequence of KDM2B wildtype (WT). The nucleotide exchanges for the corresponding amino acid changes in the relevant regions (shown in red boxes) were shown.
